# Interactions between methodological and interindividual variability: How Monetary Incentive Delay (MID) task contrast maps vary and impact associations with behavior

**DOI:** 10.1002/brb3.2093

**Published:** 2021-03-22

**Authors:** Michael I. Demidenko, Alexander S. Weigard, Karthikeyan Ganesan, Hyesue Jang, Andrew Jahn, Edward D. Huntley, Daniel P. Keating

**Affiliations:** ^1^ Department of Psychology University of Michigan Ann Arbor MI USA; ^2^ Addiction Center Department of Psychiatry University of Michigan Ann Arbor MI USA; ^3^ The Functional MRI Laboratory University of Michigan Ann Arbor MI USA; ^4^ Survey Research Center Institute for Social Research University of Michigan Ann Arbor MI USA

**Keywords:** Approach, Avoidance, fMRI, Measurement, Monetary Incentive Delay, Prediction Error, Reward Processing

## Abstract

**Introduction:**

Phenomena related to reward responsiveness have been extensively studied in their associations with substance use and socioemotional functioning. One important task in this literature is the Monetary Incentive Delay (MID) task. By cueing and delivering performance‐contingent reward, the MID task has been demonstrated to elicit robust activation of neural circuits involved in different phases of reward responsiveness. However, systematic evaluations of common MID task contrasts have been limited to between‐study comparisons of group‐level activation maps, limiting their ability to directly evaluate how researchers’ choice of contrasts impacts conclusions about individual differences in reward responsiveness or brain‐behavior associations.

**Methods:**

In a sample of 104 participants (Age Mean = 19.3, *SD* = 1.3), we evaluate similarities and differences between contrasts in: group‐ and individual‐level activation maps using Jaccard's similarity index, region of interest (ROI) mean signal intensities using Pearson's *r*, and associations between ROI mean signal intensity and psychological measures using Bayesian correlation.

**Results:**

Our findings demonstrate more similarities than differences between win and loss cues during the anticipation contrast, dissimilarity between some win anticipation contrasts, an apparent deactivation effect in the outcome phase, likely stemming from the blood oxygen level‐dependent undershoot, and behavioral associations that are less robust than previously reported.

**Conclusion:**

Consistent with recent empirical findings, this work has practical implications for helping researchers interpret prior MID studies and make more informed a priori decisions about how their contrast choices may modify results.

## Graphical abstract

This manuscript systematically evaluates common task contrasts of the Monetary Incentive Delay task and considers within‐study differences of mean‐level (or group level) activation maps, their regional associations, and effectiveness in predicting substance use and socioemotional behaviors in young adults. Our findings demonstrate more similarities than differences between positive and negative cues during the anticipation contrasts, dissimilarity between some positive anticipation contrasts, a robust deactivation effect in the outcome phase , and ROI‐behavior associations that are less robust than previously thought.

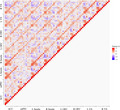

## 1 INTRODUCTION

### 1.1 Purpose

Due to the hypothesized role of reward systems in wanting, liking, and learning about rewarding stimuli, neural measurements of reward processing have become a central focus in the study of various psychopathologies and problem behaviors (Berridge & Robinson, 2003; Ernst & Luciana, 2015). The Monetary Incentive Delay (MID) task, specifically, has been frequently used to measure neural substrates of approach and avoidance mechanisms during reward processing (Knutson et al., 2000). Univariate contrasts (e.g., Big Win versus Neutral anticipation) that index neural activation during different stages of the MID task have been widely employed to study dysfunction in reward‐related processes and various maladaptive behaviors (Balodis & Potenza, 2015; Dugré et al., 2018). More recently, the task has been incorporated into large‐scale longitudinal studies to index developmental changes in reward mechanisms and their links with negative behavioral outcomes (Case y et al., 2018; Schumann et al., 2010). Despite frequent use of univariate contrasts from this task, there are relatively few studies that have examined how methodological choices made by investigators (e.g., researcher degrees of freedom), such as contrast choice, may impact the underlying results and interpretations about their findings. Therefore, this study aims to clarify the interaction between methodological and interindividual variability in MID task contrast maps and how these interactions affect their associations with psychological measures including substance use and socioemotional functioning.

### 1.2 The MID task and theories of reward processing

As of this publication, the MID task has been used in functional magnetic resonance imaging (fMRI) research for almost 20 years and is considered a robust measure of incentive motivation (Knutson & Greer, 2008; Knutson et al., 2000). As an *instrumental‐reward task*, the MID delivers rewards that are contingent on performance involving a timed button response (Richards et al., 2013), whereby different neural regions are recruited depending on whether the reward is being anticipated (i.e., wanted) or consumed (i.e., liked) (Haber & Knutson, 2010). The task was designed to localize reward‐related brain activation in substance use populations (Knutson & Heinz, 2015) and identify correlates of individual differences in positive and negative arousal (Wu et al., 2014). A central assumption of the task, inspired in part by the literature on Pavlovian conditioning (Pavlov, 1927) and dopamine responses to positive cues (Schultz, 1998), is that there are brain regions responsible for anticipating and responding to salient stimuli that have positive or negative valence. Projections from the dopamine (DA)‐rich ventral tegmental area (VTA) are thought to enhance activation in striatal regions that respond to reward anticipation (e.g., tones or cues that predict incentives) and in mesial prefrontal regions that respond to reward outcomes (Breiter et al., 1996; Knutson et al., 2000). The task allows a comparison of valence (positive valence, such as winning, or negative valence, such as losing, across big, or small rewards) and temporal phase (anticipation or outcome).

Activation patterns within anticipation and outcome phases would be expected to align with recent theories of reward processing. For instance, the first stage during cue presentation (prior to probe, or response phase) may be modeled as a “wanting” phase, eliciting motivation (or saliency of the reward cue). This *anticipation phase* should evoke robust activation in striatal regions as DA has been shown to have robust effects on wanting (or incentive salience) in both animals and humans in the ventral striatum (VS) and ventral pallidum (Berridge, 2007, 2019; Berridge & Kringelbach, 2015). However, during negative arousal (e.g., loss cue) the MID would elicit avoidance behavior which is reflected by activation in the insula (Knutson & Greer, 2008). Conversely, when modeling the *outcome phase* (or liking), one would expect less activation of VS (as only ~ 10% of neurons in nucleus accumbens facilitate pleasure) in response to the pleasure of reward. Hedonic “hot spots” are more likely to be represented in the insula and OFC (Berridge & Kringelbach, 2015) which are reported to be modulated by opioid receptors (Berridge et al., 2010; Buchel et al., 2018; Korb et al., 2020).

It is notable that the specific univariate contrasts used to index reward‐related psychological constructs often vary considerably between studies (see Supplemental Table S2). In cases of wanting rewards, reward anticipation is operationalized using contrasts such as All Win versus Neutral (Bourque et al., 2017; Martz et al., 2018; Xu et al., 2017), Big Win versus Neutral (Cao et al., 2019; Cope et al., 2019; Papanastasiou et al., 2018), or Big Win versus Small Win cues (van Hulst et al., 2015; Martz et al., 2016; Stevens et al., 2018). Likewise, in the case of reward consumption, reward outcome is operationalized using contrasts such as Reward Hit versus Neutral Hit (Chan et al., 2016; Mikita et al., 2016; Swartz et al., 2019) or Reward Hit versus Reward Miss cues (Mikita et al., 2016; Navas et al., 2018; Richards et al., 2016). The use of different contrasts to probe the same reward‐related constructs is one major source of variability in the MID literature.

The vast majority of fMRI analyses using the MID task focus on specific, unmodulated phases of the task. However, previous work suggests that modulators based on formal models of reinforcement learning may be important to incorporate into the task to account for individual variability not captured in standard subtraction analysis (Bjork et al., 2010; Oldham et al., 2018). Although reinforcement learning models have been successfully applied to the MID task (Cao et al., 2019), the utility of prediction error is still debated (Berridge & O’Doherty, 2014) and it remains to be seen how expected value and prediction error model parameters (positive or negative) modulate the signal in the anticipation and outcome phases during the MID task. Such modulators may be critical in accounting for individual‐level variation that drives performance and learning values that may be represented in subcortical and cortical neural signatures (Balleine & O’Doherty, 2010). As contingencies in the MID are based on performance, and therefore relatively uncertain, the MID differs from traditional reinforcement learning paradigms used to investigate prediction errors as the expectancies are less reliable. Therefore, the MID task may be considered a proxy to a true temporal‐difference learning task that engenders more reliable expectancies. Nonetheless, previous work has recommended the use of modulators in the MID task (Bjork et al., 2010; Oldham et al., 2018), and recent studies have found that prediction error was positively related to activation in the bilateral VS (Cao et al., 2019) and substance use problems in young adults (Cao et al., 2020) during the MID.

### 1.3 Differential use and researcher degrees of freedom in mid task

Although the MID task has been used extensively to study dysfunctional reward processing in populations with substance use disorders (Balodis & Potenza, 2015), it has also been incorporated into other studies of neurodevelopment and broader psychopathology. Various versions of the MID task have been used to investigate reward‐related changes as a function of age (Bjork et al., 2010; Dhingra et al., 2019; Heitzeg et al., 2014), social versus nonsocial rewards (Schwartz et al., 2019), psychosocial characteristics of impulsivity and sensation seeking (Büchel et al., 2017; Cao et al., 2019; Joseph et al., 2016), early adversity (Boecker et al., 2014; Gonzalez et al., 2016), substance use (Aloi et al., 2019; Cope et al., 2019; Heitzeg et al., 2014; Karoly et al., 2015; Nestor et al., 2019; Sauder et al., 2016; Swartz et al., 2019), depression (Chan et al., 2016; Colich et al., 2017; Landes et al., 2018; Mori et al., 2016), and other psychiatric symptoms (Bourque et al., 2017; Lancaster et al., 2016; Maresh et al., 2019; Mikita et al., 2016; Papanastasiou et al., 2018; von Rhein et al., 2015; Stevens et al., 2018; Urošević et al., 2016; Veroude et al., 2016; Xu et al., 2017). Across these studies, a wide range of brain‐behavior effects are reported. In addition to using different versions of the MID task, the studies cited above often used different univariate contrasts to derive activation maps. This raises the question: “To what extent do analytic methods, such as variation in univariate contrast selection, inform differences and/or similarities in conclusions about psychological characteristics?”.

Empirical evidence suggests that analytic decisions may result in substantially different interpretations of fMRI analyses. Carp (2012) demonstrated that the analytic flexibility in fMRI can generate thousands of statistical maps that can be used in subsequent analyses. As shown by Botvinik‐Nezer et al. (2020), the level of flexibility in task‐based fMRI analyses can produce different outcomes even when researchers start with identical data and hypotheses. Specifically, seventy different teams analyzed identical fMRI data with predefined hypotheses regarding risky decision‐making. Despite the similarities across data and hypotheses, between laboratory differences in contrast selection and region of interest specification altered the interpretation of results. Thus, without a clear understanding of how analytic decisions impact our results and interpretations, the flexibility of fMRI analyses (e.g., “researcher's degrees of freedom”) may result in an unacceptable number of false positives (Gelman & Loken, 2014).

In the MID task, it is not well understood how investigators’ analytic choice of contrasts (e.g., defining anticipation of reward as follows: $5 Win Cue versus Neutral Cue, or both Win Cues [$5 & $0.20] versus Neutral Cue) may impact their inferences about the association between the neural response to reward and behavior. FMRI activation maps differ as a function of reward type/magnitude (Bartra et al., 2013; Bjork et al., 2010), and recent reviews suggest there is substantial variability across studies in the techniques used to derive such maps (Balodis & Potenza, 2015; Dugré et al., 2018; Oldham et al., 2018). Contrast selection is important to the interpretation of the reported effects because experimental and baseline conditions are hypothesized to reveal components of a cognitive process which are reflected in the neural activation (Caplan, 2007). Yet, different reward contrasts, such as Big Win versus Neutral or Big Win versus Small Win cues, may be used interchangeably to operationalize reward anticipation. Combined with publication biases, the diverse sets of analyses may contribute to underreported contrasts and associations with behavior that may relate to the arbitrary decisions in the analytic pipeline (Simmons et al., 2011). Therefore, it is important to quantify how univariate contrast‐related variation in activation maps within a given sample influences the relative utility of these maps for predicting behavioral outcomes. This would demonstrate whether there is a) stability within estimates of activation at each phase of the task (anticipation or outcome); b) consistency between conceptually related contrasts in the level of activation in specific regions of interest (ROI; such that there is higher correlation within win than between win and loss anticipation); and c) whether choice between contrasts that, in theory, probe a shared cognitive process, such as anticipating rewards, alters associations between neural activation, and a psychological characteristic.

This would be difficult to deduce from a meta‐analysis for several reasons. First, meta‐analyses typically assess spatial overlap between contrasts and/or assess relations between different contrast activations and external covariates (e.g., behavioral scales or clinical disorders), but do not assess whether activations from these contrasts represent distinct versus largely overlapping individual difference dimensions. Second, most empirical studies report a constrained number of MID contrasts, while in some cases making post hoc justifications for why a particular contrast, or set of contrasts, was included in the paper. Hence, conclusions from meta‐analyses obfuscate the influence of researcher degrees of freedom linked to contrast choice and selective reporting.

### 1.4 Current study

Previous reviews of the MID task have evaluated general utilization of the task in studies of reward responsiveness (Lutz & Widmer, 2014), between‐study, temporal, and phase‐related differences in MID activation effects (Oldham et al., 2018), dynamics of reward versus loss (Dugré et al., 2018), and influences of substance use (Balodis & Potenza, 2015) and psychosis profiles (Radua et al., 2015) on activation differences. However, the extent to which contrast choice contributes to variability in activation maps, impacts the measurement of behaviorally relevant individual difference dimensions, and alters conclusions about associations between neural responses and behavior is still unclear. The current study leverages a community sample of late adolescents/emerging adults to examine variability across various univariate contrast activation maps in the MID task.

In order to delineate variability across contrast types (which is difficult to evaluate between samples/studies), we perform multiple common analyses that focus on the anticipation, outcome, and prediction error parameters, with data from the same individuals. Due to the a) prominent role of motivation (or anticipation of reward) in this task; b) the critical role of dopamine in anticipation (“wanting”) and not outcome (“liking”) (Berridge & Kringelbach, 2015); c) difficulty to temporally differentiate the outcome phase (Bjork et al., 2010); and d) the drop in power during the outcome phase as each anticipatory trial is split into “hit” or “miss” trial outcome, 50% of contrasts focus on the anticipation phase of the MID task. These activation maps are thresholded to compare the degree to which statistical maps (from ten contrasts) a) vary within a phase (e.g., anticipation Big Win > Neutral versus Big Loss > Neutral contrasts) and b) vary between phases of the task (e.g., anticipation versus outcome). The degree of variability is assessed at the individual level and group level to quantify the general pattern in overlap of active voxels between two given contrast's activation maps. Then, mean signal intensity values for key regions from previous reviews, such as the insula, mPFC, OFC, VS, and amygdala (Balodis & Potenza, 2015; Dugré et al., 2018; Oldham et al., 2018), are extracted to evaluate whether activation in these ROIs from different contrasts index convergent or divergent dimensions of cognitive processing (such as reward anticipation). Finally, Bayesian correlations between these ROI mean signal intensities and self‐reported measures are assessed to determine the impact of contrast choice on the association with psychological measures including substance use, psychosocial, and socioemotional functioning.

While meta‐analyses have proposed region‐specific activations for positive and negative values across fMRI tasks (Bartra et al., 2013), a recent review of the MID yielded overlapping networks across positive and negative values (Oldham et al., 2018). Given the within‐sample comparison of contrasts, instead of testing specific hypotheses within a null hypothesis significance test framework in these analyses, similarities and differences are presented as an index of overlap (Jaccard's similarity coefficient), and statistical association across ROIs and behavior (Pearson's *r* coefficient; heat maps of *r* point estimates for inter‐ROI relationships; and posterior distributions of *r*‐values for associations of ROIs with behavioral covariates). Our broad goal is to improve the field's understanding of how and where there is within‐task variability as a function of MID task contrast choice, and, in doing so, to inform the interpretation of existing MID studies and better guide researchers’ a priori decisions about which specific contrasts the hypotheses are based on in future studies. This exploratory analysis can provide inferences about how contrast selection, which typically precedes the reporting of results and increases researcher degrees of freedom, affects the activation maps. Due to the exploratory nature of the analyses, the background, methods, and analytic plan were preregistered on the Open Science Framework (https://osf.io/xh7bz). However, we elected not to preregister specific hypotheses related to brain‐behavior associations because the intended purpose of the study was to use exploratory analyses to provide a holistic overview of how researcher degrees of freedoms impact interpretation of MID task results (Thompson et al., 2020).

## 2 METHODS

Participants in this neuroimaging study are from a subsample of the Adolescent Health Risk Behavior (AHRB) study. AHRB consists of a nonprobability sample of 2,017 (Age Mean = 16.8, *SD* = 1.1; Female 56%) 10th‐ and 12th‐grade students recruited from nine public school districts across eight Southeastern Michigan counties, using a quota sampling method to enhance sample diversity. Phase I, described elsewhere (Demidenko et al., 2019), collected demographic, psychosocial, neurocognitive, and behavioral information across three waves of survey data collection. From Phase I of the study, a subsample of 115 adolescents, who were characterized as high and average/low risk, was recruited to participate in the neuroimaging phase of the study (elapsed time between Wave 1 and neuroimaging section (Months): *M* = 30.9 months *SD* = 5.0 months). Of the 115 adolescents that participated, 108 completed the magnetic resonance imaging (MRI) portion of the visit. Seven participants were ineligible or unable to participate in the MRI due to not meeting MRI safety eligibility. Of the 108 participants that completed the MRI, four participants were excluded from the analyses due to: artifacts in the images that were not recoverable, and one participant that stopped responding during the second run of the task. The final fMRI subsample (*N* = 104; Age Mean = 19.3, *SD* = 1.3; Female 57%) was included in the subsequent analyses and did not differ from the full sample in age, gender, or time from the original survey. The bulk of code used in the subsequent analyses is made available online (https://github.com/demidenm/MIDContrasts).

### 2.1 Self‐reported psychological measures

#### 2.1.1 Substance use

Substance use behaviors (marijuana and alcohol) are assessed via the item: “On how many occasions (if any) have you [used marijuana or hashish/had any alcoholic beverage to drink—more than just a few sips] during the last 12 months?”. Responses are reported on a seven‐point scale ranging from (1) = “0 occasions” to (7) = “40 or more occasions.” Substance use items are identical to those used in the annual, national Monitoring the Future surveys (Johnston et al., 2019). Marijuana and alcohol scores were z‐scored, and then, a substance use aggregate measure was created by averaging the z‐scored items across Wave 1 – Wave 3.

#### 2.1.2 Impulsivity

The Barratt Impulsiveness Scale‐Brief (BIS‐B) is an 8‐item, unidimensional measure of impulsiveness (Steinberg et al., 2013) based on a reduced item set obtained from the Barratt Impulsiveness Scale (BIS), 11th revision. Items were rated on a 4‐point Likert‐type scale: (1) = rarely/never, (2) = occasionally, (3) = often, and (4) = almost always/always. A mean score was computed (range: 1 – 4), higher scores indicated lower self‐reported impulsivity (α = 0.79). BIS‐B items were z‐scored and then aggregated by averaging scores across Wave 1 – Wave 3.

#### 2.1.3 Sensation seeking

The Brief Sensation Seeking Scale (BSSS) is an 8‐item self‐report measure of sensation seeking (Hoyle et al., 2002) based on a reduced item set of the Zuckerman Sensation Seeking Scale (SSS). The items measure dimensions of sensation seeking: experience seeking, boredom susceptibility, thrill and adventure seeking, and disinhibition. Responses were on a 5‐point Likert scale: (1) = strongly disagree, (2) = disagree, (3) = neither disagree nor agree, (4) = agree, and (5) = strongly agree. A mean score was computed (range: 1–5), with higher scores indicated higher self‐reported sensation seeking (α = 0.78). BSSS items were z‐scored and then aggregated by averaging scores across Wave 1 – Wave 3.

#### 2.1.4 Socioemotional problems

Socioemotional problems were assessed using the Youth Self‐Report (YSR; Achenbach & Rescorla, 2001) to characterize externalizing and internalizing problems. The YSR is a widely utilized, 112‐item self‐report measure assessing emotional and behavioral difficulties in 11‐ to 18‐year‐olds. The YSR includes two broadband scales: internalizing problems (e.g., withdrawn/depressed) and externalizing problems (e.g., attentional deficit/hyperactivity problems, oppositional defiant problems). Raw scores are normalized to provide a common metric with higher scores indicating greater psychopathology. Validity and reliability of the YSR broadband, syndrome, and DSM‐oriented scales are well documented (Achenbach, 2013; Achenbach & Rescorla, 2001) with adequate internal consistency (α = 0.70 ‐ 0.86) and test–retest reliability (α = 0.67 ‐ 0.88). In the present study, Cronbach's alphas of 0.91 and 0.88 were obtained for the internalizing and externalizing scales, respectively. An aggregate score was created from population‐standardized z‐scores for internalizing and externalizing by averaging scores across Wave 1 – Wave 3.

### 2.2 fMRI task

A modified version of MID task (Knutson et al., 2000) was used to model neural signatures of the anticipation and outcome of monetary rewards. The modified version in this study is currently being employed in the national Adolescent Brain Cognitive Development (ABCD) study to measure the development of adolescent reward processing (Case y et al., 2018). Identical to the task described in Case y et al. (2018), the task in this study consists of three phases: anticipation, probe, and outcome (i.e., feedback). Each trial starts with a cue type (Win $5, Win $0.20, Lose $5, Lose $0.20, or No Money At Stake). There are twelve trial orders of the task, consisting of 50 contiguous trials and 10 trial types per run (5 min 42 s long). Participants completed two runs of the MID task during the scan (100 trials and 20 trial types). The task is individualized to reach around 60% accuracy rate by adjusting the difficulty (i.e., probe duration). See Section 1.1 in Supplementary Materials for more information on task paradigm and administration. A key difference between the current version of the MID (and the one used in the ABCD study) and that used in the IMAGEN sample (Cao et al., 2019) is the IMAGEN study only includes Win and Neutral trials, thus excluding Loss trials. Furthermore, in the IMAGEN, study performance was rewarded with “points” that were exchanged for M&M’s/candy in contrast to a concrete reward for task performance (e.g., money).

### 2.3 fMRI data acquisition and preprocessing

Data were acquired using a GE Discovery MR750 3.0 Tesla scanner with a standard adult‐sized coil (Milwaukee, WI). A full‐brain high‐resolution T1 SPGR PROMO scan was acquired that is used in preprocessing (TR = 7,000 ms, TE = 2,900 ms, flip angle = 8°, FOV = 25.6 cm, slice thickness = 1 mm, 208 sagittal slices; matrix = 256x256). Before the MID task, a fieldmap was acquired using spin‐echo EPI (TR = 7,400 ms, TE = 80 ms, FOV = 21.6 cm, 90x90 matrix) with opposite phase encoding polarity (A➔P, P➔A). Two functional T2*‐weighted blood‐oxygen‐level‐dependent (BOLD) MID runs were acquired in the axial plane following structural and a face task using a multiband EPI sequence (MB factor = 6) of 60 contiguous axial 2.4 mm slices (TR = 800 ms, TE = 30 ms, flip angle = 52°, FOV = 21.6 cm, 90x90 matrix, volumes = 407).

### 2.4 fMRI data analyses

FMRI data were reconstructed; realignment and fieldmap correction was applied in SPM12 to each T2* run to recover inhomogeneity of signal in the B0 field, and physiological noise was removed using RETROICOR (Glover et al., 2000). Preprocessing steps were completed using FSL (FMRIB's Software Library, www.fmrib.ox.ac.uk/fsl) FEAT (FMRI Expert Analysis Tool) version 6.00. After volumes were (1) reconstructed, (2) realigned, (3) physiological noise was removed, and (4) field map correction was applied, the following preprocessing steps were performed: (5) registration to high‐resolution structural and standard space MNI 152 image using FLIRT using a Full search 12 DOF (Jenkinson et al., 2002; Jenkinson & Smith, 2001), (6) motion correction using MCFLIRT (Jenkinson et al., 2002), (7) nonbrain removal using BET (Smith, 2002), (8) spatial smoothing using a Gaussian kernel of FWHM 5 mm, (9) grand‐mean intensity normalization of the entire 4D data set by a single multiplicative factor, and (10) high‐pass temporal filtering (Gaussian‐weighted least‐squares straightline fitting, with sigma = 50.0 s).

## 3 fMRI ANALYSES

Subjects were excluded from analyses if a subject's mean framewise displacement (FD) values exceeded > 0.9 within any given run (mean FD pre‐ and post‐preprocessing included in Supplementary Section 1.2), all subjects’ mean post‐FD was < 0.9. We focused on commonly used contrasts (Table 1) from a recent review (Oldham et al., 2018) and those from our review of studies using the MID (PubMed 2015 – 2019; Supplementary Table S2), such as reward anticipation (such as Big Win ($5) or All Win ($5 & $0.20) versus Neutral anticipation), Win outcome hit (such as $5 versus Neutral hit outcome, loss conditions (such as $5 or $0.20) and alternative contrasts that may be comparable to test for similarities within a group, for example, win or big win conditions. It should be noted that using anticipation versus outcome phase yields estimates that are often powered differently, as a function of the target accuracy of the task (60%) leading to individual variation in hit/miss trials. Furthermore, since the outcome phase is often difficult to deconvolve in the task and modeled in various ways (see Supplementary Table S2 **)**, we include one type of outcome contrast focusing on gain and loss, as it is not a central focus of these analyses and often not the focus in contrasts in the literature.

**TABLE 1:** Contrast modeled in the monetary incentive delay task

Contrasts	Phases of MID Modeled
Contrast 1 (A1) ‐ Ant	Win (W; $5 & $0.20) > Neutral (N) (W > *N*)
Contrast 2 (A2) ‐ Ant	Big Win (BW; $5) > Neutral (N) (BW > *N*)
Contrast 3 (A3) ‐ Ant	Big Win (BW; $5) > Small Win (SW; ($0.20) (BW > SW)
Contrast 4 (A4) ‐ Ant	Big Win (BW; $5) > Implicit Baseline (BW > IB)
Contrast 5 (A5) ‐ Ant	Big Loss (BL; $5) > Neutral (N) (BL > *N*)
Contrast 6 (O6) – Out	Big Win (BW; $5) Hit > Neutral (N) Hit (BWH > NH)
Contrast 7 (O7) – Out	Big Loss (BW; $5) Hit > Neutral (N) Hit (BWH > NH)
Contrast 8 (P8) ‐ PE	Expected Value – BW & SM Modulated (EV)
Contrast 9 (P9) ‐ PE	Positive Prediction Error (PPE) ‐ BW & SM Modulated
Contrast 10 (P10) ‐ PE	Negative Prediction Error (NPE) ‐ BL & SL Modulated

Note

Ant = anticipation; Out = outcome; individual contrasts modeled in FSL, see section 1.3 in Supplementary for list of events modeled in GLM. A = anticipation; O = Outcome; PE = prediction error

First‐level analyses were performed by using FEAT. Time‐series statistical analysis was carried out using FILM with local autocorrelation correction (Woolrich et al., 2001). Similar to other studies (Cao et al., 2019; Hagler et al., 2019; Lamm et al., 2014), both anticipation and outcome events were modeled (15 explanatory variables) and modulated prediction error signal of EV, PPE, and NPE (see Table 1), in addition to six motion parameters (translations and rotations in x, y, z directions) and the derivatives of the motion parameters. The modeled contrasts and design matrix are described in greater detail in Supplementary Section 1.3. We included prediction error explanatory variables based on a recent review, suggesting the MID is considered to be an implicit reinforcement learning (RL) paradigm (Balodis & Potenza, 2015), and others recommending use of modulators (Bjork et al., 2010; Oldham et al., 2018). However, as noted in the introduction, the MID is not a true RL design but only a proxy. To incorporate these recommendations, the RL modulators included the following: expected value (EV) and prediction error (PE). To derive estimates of EV and PE for this task, the behavioral data were modeled for each participant (100 trials – trial‐by‐trial) to calculate parametric modulators (EV for anticipation; PE for received reward (RR); *pGain* = probability gain, η = learning rate (0.7)). Similar to Cao et al. (2019), we used a RL model trained by reward cues and outcomes (Rescorla & Wagner, 1972):

$$EV_{t}=pGain_{t}\times Cue_{t}$$

$$PE_{t}=RR_{t}\times EV_{t}$$

$$pGain_{t+1}=pGain_{t}+\left(\eta \times \frac{PE_{t}}{Cue_{t}}\right)$$

To average across the two runs that are used in subsequent stages, a second‐level model was defined for each participant for each of the ten contrasts (see Supplementary Section 1.3 **)** using fixed effect analysis in FEAT. A group‐level analysis was performed using FMRIB’s Local Analysis of Mixed Effects (FLAME 1) to generate a mean‐level activation across subjects for a given contrast. Considering the large array of contrasts that are modeled, abbreviations from the first column of Table 1 are referred to when referencing contrasts henceforth.

To provide a direct observation of the BOLD signal and signal‐to‐noise information of subcortical regions, we include complementary post hoc analyses evaluating raw BOLD signal (see Section 2.8 in Supplemental Materials). We extract the mean signal for VS and mPFC in the time‐series for VS and plot it for 15 TRs. Likewise, for cortical mPFC and subcortical VS we extract and present the distribution of the signal‐to‐noise ratios (SNR) for each individual and run to confirm that SNR is within an acceptable range (see Section 2.5 in Supplemental Materials).

### 3.1 Individual level and group estimates

In order to compare overlap between thresholded activation maps for each contrast at the individual level and group level, we thresholded activation maps produced by the second‐level and group‐level analyses. For the individual level, subjects' second‐level maps (zstat) for each contrast are thresholded at *p* <.01 (*z* = 2.3) and group‐level contrasts are thresholded at *p* <.001 (*z* = 3.1). We selected a lower threshold for individual maps due to more variability in estimates within an individual map that may substantially alter Jaccard's Similarity Indices. These thresholded maps are binarized (using *fsl* ‐bin) and compared to derive Jaccard's Similarity Indices (described below).

### 3.2 Calculating similarity

One of the aims for this study is to compare similarity, or spatial overlap, between different activation maps of the MID task within individuals and at the group level. This is to provide an easy to interpret index of how similar (or different) activations are across contrast types. Similar to a previous work (Grady et al., 2020), we calculate a percent overlap using Jaccard's similarity index (JSI) (Maitra, 2010) between contrasts. The JSI calculates the number of voxels that overlap across two thresholded statistical maps. One of the major advantages of using the JSI is that the percent overlap results obtained from this technique are intuitive and physically interpretable (Maitra, 2010). The percent overlap between any two activation maps is defined from a set theoretical point of view, where the overlap $J\left(A,B\right)$ is defined by the well‐known relation as follows:

$$J\left(A,B\right)=\frac{A\cap B}{A\cup B}$$

As we used JSI point estimates to evaluate activated voxels across different thresholded contrasts, we propose a bootstrapping‐based confidence interval calculation for identifying the 95% confidence intervals of the overlap measures across all subjects in our sample (DiCiccio & Efron, 1996). The bootstrapped JSI would provide reliable estimates of the range and shape of the distribution of percent overlap and a physical interpretation of the JSI obtained across all of the subjects. Although the thresholded maps are impacted by power in the design, similarity can be assessed within phases, such as anticipation or outcome, given the number of trials is comparable within each phase (with the exception of the all win contrast).

### 3.3 Region of interest and behavioral associations

Central voxel coordinates from Neurosynth.org for a priori ROI’s: bilateral insula, OFC, VS, and mPFC and ACC (see Supplemental Table S1 and Figure S1), were used to create 10‐mm‐diameter spheres. For each ROI, the voxels from each contrast mask (using z‐statistics produced by feat second level) are averaged to create a mean signal intensity value and extracted using *fslmeants*. Correlations (point estimates of Pearson's *r*) across ROIs were analyzed in R version 3.6.1 (R Core Team, 2019) and were visualized using a heatmap.

ROI mean‐level signal intensity values across ten contrast types (described above) were used to assess associations between neural activity and self‐reported aggregate z‐scores of a) substance use, b) sensation seeking, c) impulsivity, d) externalizing, and e) internalizing problems. Bayesian correlation analyses implemented in JASP (JASP Team, 2019; Ly et al., 2018) were used to estimate posterior distributions for the Pearson's *r* value of each predictive association. Default, noninformative priors (uniform distributions spanning the values from −1 to 1) were used for all correlation analyses. Median values of the posterior distribution, which indicate the most likely *r* value, and 95% credible intervals, which represent the lower and upper bounds of the range which has a 0.95 probability of containing the *r* value, are reported below to quantify the strength of, and uncertainty about, these predictive associations. As analyses are not intended to be formal tests of hypotheses, we will refrain from reporting either Bayes factors or frequentist *p*‐values.

## 4 RESULTS

### 4.1 Demographics, task behavior, and general overview

The demographic characteristics for the full sample (*N* = 104) are provided in Supplementary Section 2.2, Table S3. For the anticipation phase (A1‐A5) and prediction error models (P8‐P10), all 104 individuals were included (Note: We remind the reader to refer to Table 1 for contrast descriptions). However, for the outcome phase (O6 & O7) four subjects were excluded due to underpowered conditions resulting in anomalies in the estimated [first level & second level] statistical maps, resulting in *N* = 100 for the outcome contrasts. The behavioral performance statistics from the MID task are included in Supplementary Section 2.3, Table S4 and Figure S2. Although the average accuracy for the task, 57%, was below the targeted 60%, the Big Win ($5) and Big Loss ($5) conditions were at or above the target, 62% and 60% accuracy, respectively. As expected, accuracy was lower (48%) and more variable during the neutral condition. Mean response times are not reported, as the E‐Prime data were not collected for incorrect (“miss”) trials during the MID task.

**FIGURE 1:**
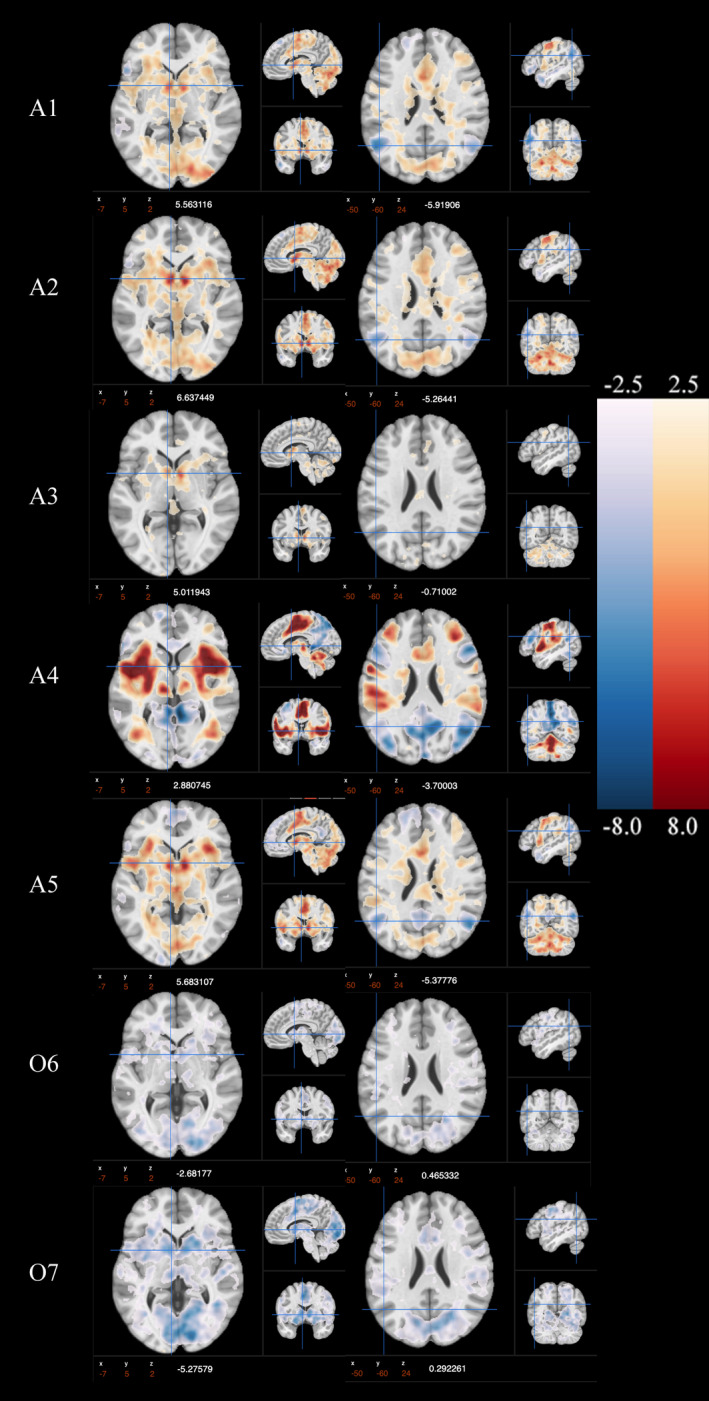
Mean level activation and deactivation maps for A1‐A5 & O6‐O7, one‐sample *t* test. See Table 1 for details and online collection for unthresholded statistical maps of tens contrasts https://neurovault.org/collections/6210/

**FIGURE 2:**
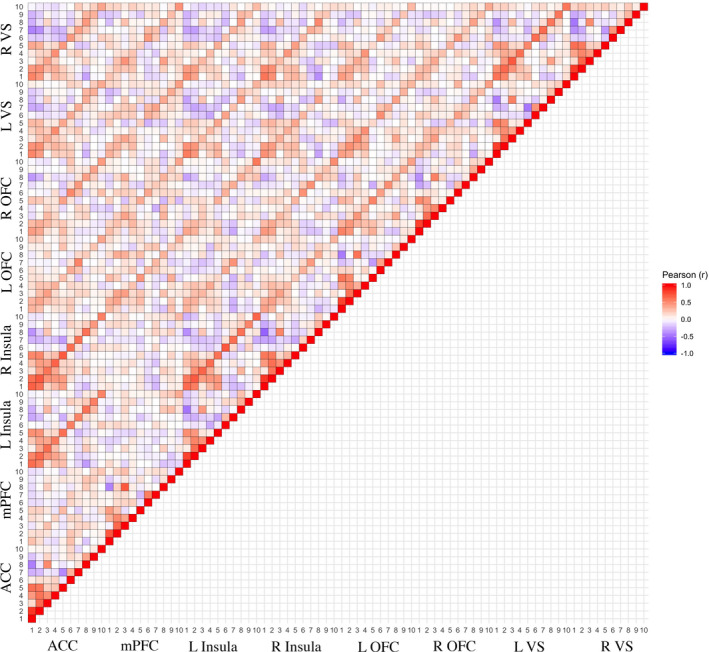
Pearson correlation matrix of 10 contrasts by 8 ROI’s. Color bar represents the associated Pearson's *r* value between the 10mm ROI across 10 contrasts. See Table 1 for associated contrast information. R = right; L = left; VS = ventral striatum; OFC = orbitofrontal cortex; mPFC = medial prefrontal cortex; ACC = anterior cingulate cortex

**FIGURE 3:**
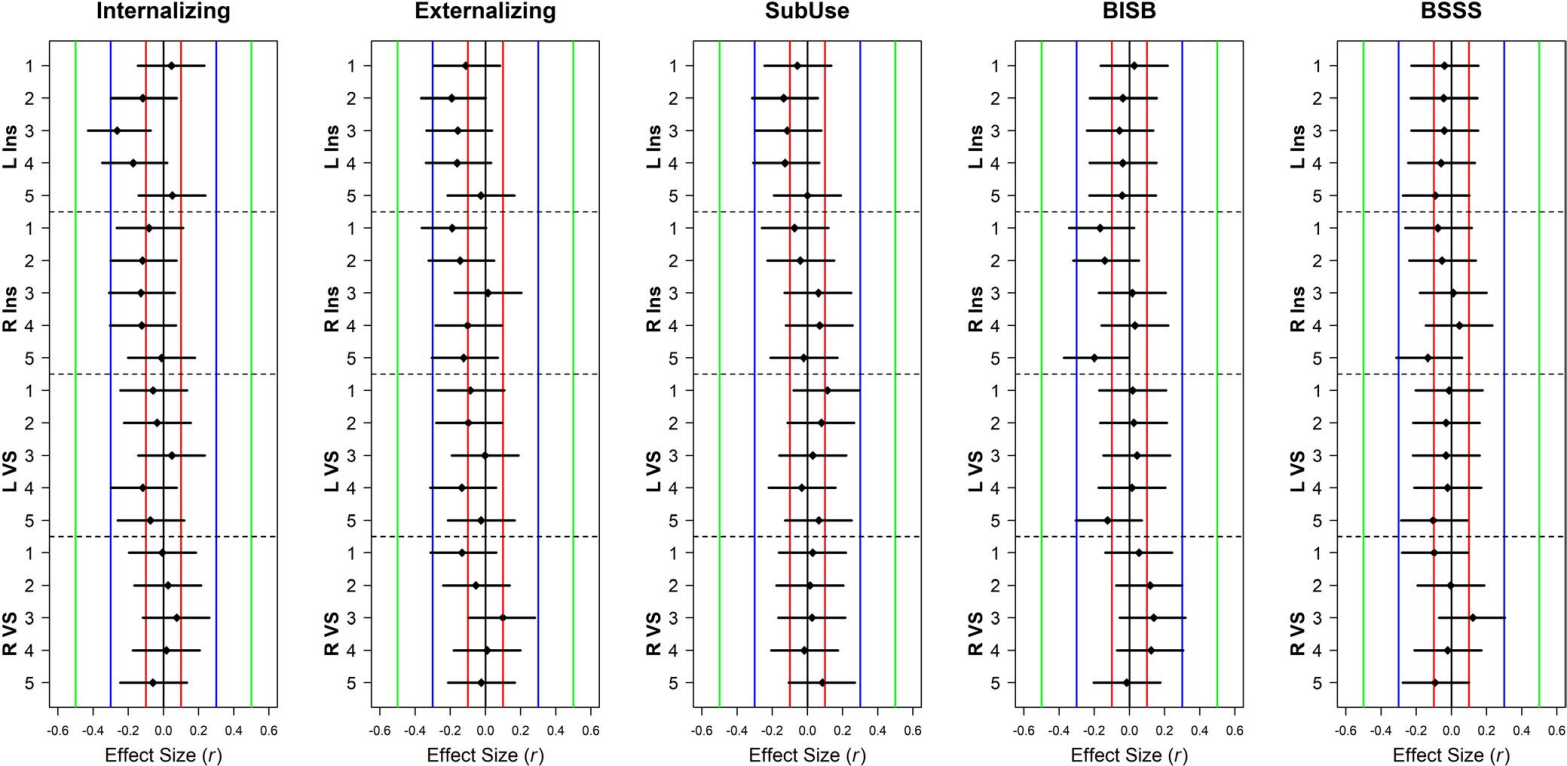
Forest plots displaying the most likely Pearson's *r* value (black diamonds) and 95% Bayesian credible interval (black lines) for correlational relationships between ROI activation estimates from each anticipatory contrast and behavioral criterion measures. Red, blue, and green lines denote “small” (r=0.10), “moderate” (r=0.30), and “large” (r=0.50) effect sizes. 1–5 = Five contrasts listed in Table 1; L = left; R = right; Ins = insula; VS = ventral striatum; SubUse = substance use composite measure; BIS‐B = Barratt Impulsiveness Scale‐Brief; BSSS = Brief Sensation Seeking Scale (behavioral items are z‐scored)

JSI similarity matrices and activation maps are displayed in Supplementary Figure S4 and Figure 1, respectively. Associations between‐individual differences in ROI mean‐level activation from each contrast are reported at https://osf.io/a5wem/ and in Figure 2 and are selectively reported below for clarity. Correlations between ROI mean signal intensity estimates and behavioral criterion measures are reported in Figure 3 (subset of four regions, five anticipatory contrasts across our five behaviors; full figure reported in Supplement Figure S5, section 2.7) and available at https://osf.io/d9k3v/. There were four notable patterns present in these results: (1) Win and Loss anticipation demonstrate comparable striatal/insula activation and task‐negative deactivation (see NeuroVault statistical map: https://neurovault.org/images/359858/); (2) outcome phase contrasts consistently imply deactivation of striatal regions (potentially due to artifact related to signal spillover); (3) the Big versus Small Win contrast appears less meaningful than, and unrelated to, other anticipation phase contrasts; and (4) individual differences in ROI activation, across different contrasts, demonstrate relatively weak associations with behavior. The aforementioned are expanded in greater detail below. Notably, the activation maps of the prediction error models were extremely variable in activation and relatively weak in their associations with mean ROI activation from other contrasts; therefore, they are not discussed below. The contrast maps are available online and results presented in Figures 2, 3.

### 4.2 Big Win and Big Loss anticipation engage similar neural regions

The thresholded masks (*p* <.001) of **A2:**BW > N and **A5:**LB > N group maps had Jaccard's similarity Coefficient of 0.16 (Supplemental Figure S4). This similarity is also apparent in the group‐level activation maps, demonstrated by shared patterns of activation (Figure 1). Although the peak left striatal activation in the **A2:**BW > N is greater than in the **A5:**BL > N (based on magnitude of *z*‐statistic in activation maps), in their direct comparison (https://neurovault.org/images/359858/) the difference is relatively small. The greatest difference between these two contrasts was increased activation in the mPFC in **A2:**BW > N as compared to **A5:**LB > N. Furthermore, contrasts **A2:**BW > N and **A5:**BL > N show similar activation of supplementary motor area (SMA), the insular cortex, thalamus, and cerebellar regions. Similar to the shared positive activation of these contrasts, they, too, share comparable deactivation in the task‐negative, angular gyrus, an effect that is not seen in the **A3:**BW > SM (Figure 1). This activation in the striatal regions and deactivation in task‐negative regions is comparable to a recent meta‐analysis (open‐source activation maps: https://neurovault.org/collections/4258/) showing similar robust patterns of activation and deactivation in both win and loss anticipation (Wilson et al., 2018).

Consistent with these similarity analyses in group‐level activation, correlations of mean signal intensity values from ROIs across **A2:**BW > N and **A5:**BL > N (Figure 2, full matrix available at https://osf.io/a5wem/) also suggested that neural responses from these contrasts index similar individual difference dimensions. Positive correlations in neural responses between the contrasts were identified (Figure 2) in the anterior cingulate cortex (ACC; *r* = 0.58), medial prefrontal cortex (mPFC; *r* = 0.26), bilateral insula (Right: *r* = 0.57; Left: *r* = 0.44), bilateral orbitofrontal cortex (OFC; Right: *r* = 0.43, Left: *r =* 0.50), and bilateral ventral striatum (VS; Right: *r* = 0.57, Left: *r* = 0.49). The similarity between **A2**:BW > N and **A5**:BL > N is consistent with a recent meta‐analyses (Oldham et al., 2018).

### 4.3 Reward and Loss outcome is paradoxically linked to striatal deactivation

Contrary to past work focused on striatal activation during win conditions, our contrasts during outcome phase, **O6:**BWH > NH & **O7:**BLH > NH, demonstrated a *deactivation* of the striatal regions. Based on Jaccard's similarity Coefficient, 0.34, the regions that were deactivated were comparable in **O6:**BWH > NH and **O7:**BLH > NH (Figure 1, and Supplemental Figure S4). Although the mean‐level deactivation of the striatal region in the **O6:**BWH > NH contrast was relatively weak (*t* = −2.68), in the **O7**:BLH > NH condition the deactivation was relatively robust (*t* = −5.8). As a control comparison in change of activation, we reference the angular gyrus, which has a relatively weak mean‐level activation in both **O6:**BWH > NH and **O7:**BLH > NH, demonstrating that there is a more profound change in activation in the striatal region between the anticipation and outcome phase (see Figure 1). In a direct comparison of **O6:**BWH > NH & **O7:**BLH > NH (https://neurovault.org/images/359858/), **O6:**BWH > NH demonstrates greater activation in the left parahippocampal (*z* = 4.3) and right nucleus accumbens (*z = *3.4). These two outcome contrasts demonstrated some associations (Figure 2) in individual difference analyses of mean signal intensity in the ACC (*r* = 0.33), mPFC (*r* =0.55), and bilateral VS (left *r* = 0.45; right *r* = 0.46). Notably, this deactivation is likely to be a function of the spillover from the anticipatory phase given the short interval between anticipation and outcome stimuli, as can be observed in the BOLD signal change in Figure S6.

### 4.4 Anticipation Big Win versus Small Win contrast is distinct from other anticipation contrasts

Despite its variable use in the literature, **A3:**BW > SM was unique when compared to other contrasts in anticipation phase (Figure 1). The **A3:**BW > SM had the lowest Jaccard coefficient with other contrasts modeling the anticipation phase, <0.02 (Figure S4). Further, in the group‐level activation, compared to **A1:**W > N, **A2:**BW > N, and **A5**:BL > N anticipation contrasts, the **A3:**BW > SM had the weakest group‐level striatal and insular activation, and no task‐negative activation. The task‐negative activation difference is unique, as all of the other contrasts demonstrate this profile of task‐negative activation in the anticipation phase.

However, with respect to individual differences in ROI mean‐level activation, depending on the contrast, there are similarities between **A3:**BW > SM and other contrasts. For example, the mean‐level activation between **A1:**W > N and **A3:**BW > SM is negligible: ACC (*r* = 0.15), mPFC (*r* = −0.05), bilateral insula (left *r* = 0.07; right *r* = 0.08), bilateral OFC (left *r* = 0.02; right = 0.06), and bilateral VS (left *r* = 0.06; right = 0.15). Yet, there is a strong association between **A2**:BW > N and **A3:**BW > SM in the ACC (*r* = 0.64), mPFC (*r* = 0.65), bilateral insula (left *r* = 0.63; right *r* = 0.58), OFC (left *r* = 0.60; right *r* = 0.62), and bilateral VS (left *r* = 0.59; right = 0.66). Despite the similarity discussed between **A2:**BW > N and **A5:**BL > N above in Section 4.2, there is a negligible association between ROI’s in **A3:**BW > SM and **A5:**BL > *N* (*r* = −0.11 to 0.19), which may suggest that the similarities between **A2:**BW > N and **A3:**BW > SM may arise from the shared Big Win cue in the subtraction.

### 4.5 Across contrasts, activations show only weak to negligible correlational relationships with behavioral criterion measures

The aggregated scores for psychological characteristics in this sample were associated in the expected direction (Supplementary Section 2.4, Table S5). More specifically, there was a strong positive association between internalizing and externalizing problems (*r =* 0.51), sensation seeking and impulsivity (*r =* 0.44), externalizing and substance use (*r* = 0.51), and substance use and sensation seeking (*r* = 0.36) and impulsivity (*r* = 0.23).

Figure 3 shows a subset of Bayesian correlations between ROI mean signal intensities and behavioral criterion measures (for complete figure, see Supplemental Figure S5). It shows posterior medians and 95% credible intervals (CIs) of Pearson's *r*‐values, which represent the most likely *r* value and range in which there is a 0.95 probability that the *r* value falls, respectively (full results available at https://osf.io/d9k3v/; complimentary bootstrapped values are provided at https://osf.io/dr5y2/). Although the interpretation of individual associations is complicated by the large number of tests reported in Figure S5, several general patterns are apparent. First, 71% of the most likely *r*‐values fell at or well below the threshold for what is typically considered a “small‐sized” effect, |*r*| = 0.10 (Supplemental Table S6). Similarly, the bulk of CIs also fell in this general range. In fact, there was not a single association for which the most likely *r* value indicated a “moderately sized” effect (|*r*| >= 0.30), and few CIs overlapped with this “moderate” criterion. It is also notable that only a handful of CIs (less than 5%) did not overlap with 0, suggesting that even these cases, which might be interpreted as showing promising evidence for a non‐negligible effect, may be due to multiple testing rather than reflecting true associations. Indeed, as typical Bayesian CIs do not take into account the probability that the null (*r* = 0) is true (van den Bergh et al., 2019), the effect size estimates we report are, if anything, likely to be overly optimistic. Hence, consistent with other emerging findings from large, diverse neuroimaging data sets (Nees et al., 2012; Paulus et al., 2019; Paulus & Thompson, 2019), these patterns of results suggest that direct associations of MID task activations with relevant behavioral criterion measures are less robust than what has been previously thought and that even if these associations exist, effect sizes are likely to be small.

Second, coupled with the small effects, decisions in contrasts can weaken or alter the brain‐behavior results and thus the underlying interpretation. For instance, as can be observed in Figure 3 the median *r* for the association between anticipatory win activation in the ventral striatum and sensation seeking flips from negative to positive between **A1:**W > *N* (right, *r* = −0.10) and **A3:**BW > SW (right, *r* = 0.12). This example, and the high degree of variability in median *r* between ROI and behaviors presented in Figure 3 and Figure S5, indicates that caution should be taken when selecting contrasts as they may invariably change interpretations even in the context of these small effects.

### 4.6 Post hoc analyses

In light of prior meta‐analytic comparisons of base contrasts within individuals, such as gain versus outcome phases (Knutson & Greer, 2008; Wilson et al., 2018), we compared these differences in the anticipation phase, **A2:**BW > N versus **A5:**BL > N; outcome phase, **O6:**BWH > NH versus **O7:**BLH > NH; win anticipation versus win outcome, **A2:**BW > N versus **O6:**BWH > NH; and loss anticipation versus loss gain outcome, **A5**:BL > N versus **O7**:BLH > NH. We provide these for reference online https://neurovault.org/collections/JVXLTPHC/. Notably, in a direct comparison of **the A2:** BW > N versus **A5:** BL > N group‐level activitation we find no substantial differences in VS or insula as a function of reward and loss.

**FIGURE 4:**
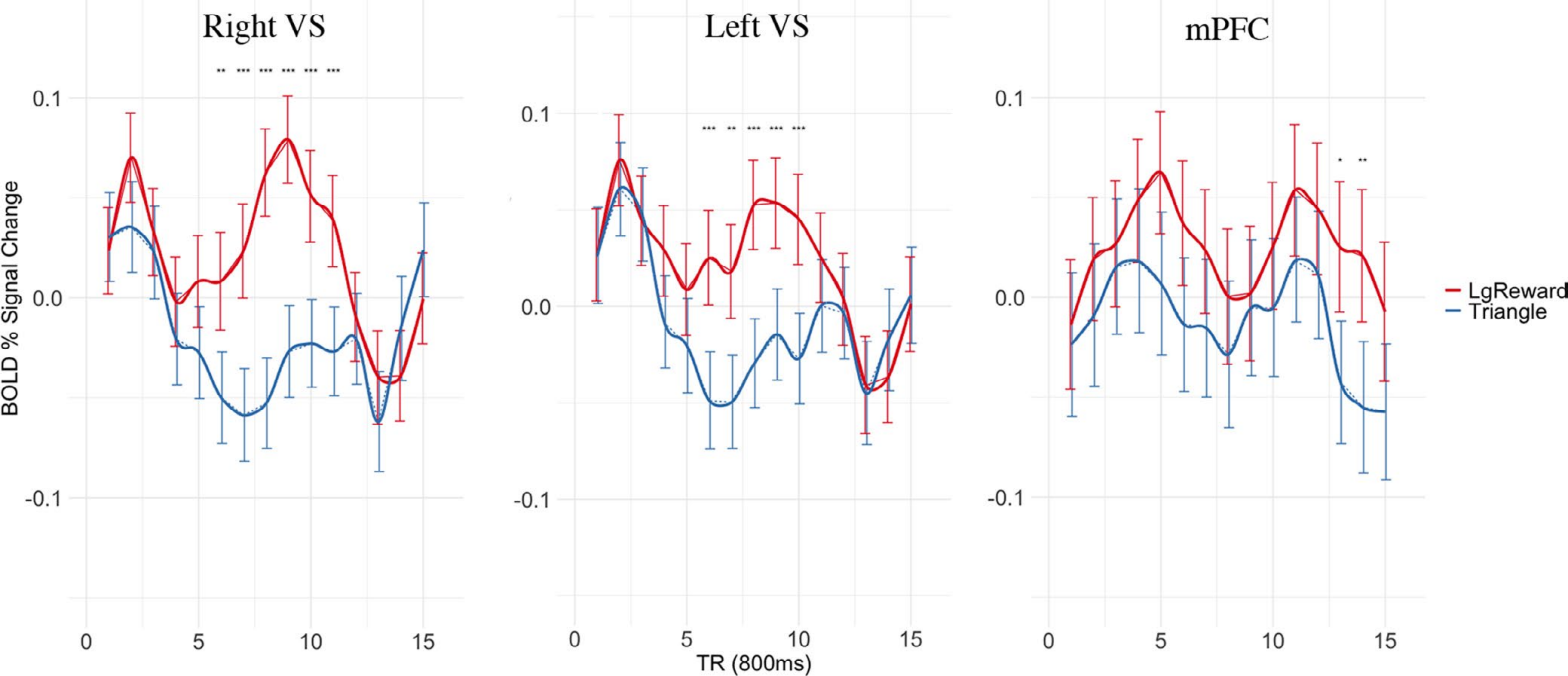
Direct observation of BOLD signal locked to cue onset for Big Win (LgReward) and Neutral (Triangle) for 15 TRs (12 s) after cue onset. mPFC = medial prefrontal cortex; VS = ventral striatum. Error bars = bootstrapped 90% confidence interval; *p* <.05*; *p* <.01**; *p* <.001**

Due to recent concerns that some multiband sequences may alter the BOLD signal in subcortical regions (Risk et al., 2018), we include signal‐to‐noise ratios and plotted time‐series from the VS to provide a direct observation of signal for each anticipation condition. With respect to the direct observation of the BOLD signal, we find appropriate separation in anticipation of Big Win and Neutral cues (Figure 4) and signal‐to‐noise ratio in the VS region (Supplementary Figure S3). With respect to the anticipation phase, we see the expected peak in BOLD separation between Big Win and Neutral cues around 7–8 s after cue onset (Figure 4). Such that, this separation is significant from TR 6 (*p* <.01) to TR 11 (*p* <.001) in the right VS, and TR 6 (*p* <.001) to TR 10 (*p* <.001) in the left VS, before the undershoot at TR 14. This separation, as expected, does not occur in the mPFC. The nature of the anticipation signal bleeding into the outcome phase is apparent in the bilateral VS when the anticipation cues are locked to the outcome phase (Supplementary Figure S9).

## 5 DISCUSSION

In this study of the MID task, we performed an evaluation of similarities and differences between commonly used univariate contrasts, focusing on spatial overlap, individual differences in mean ROI signal intensity, and correlations between ROI activations and behavioral criterion measures. After identifying ten candidate contrasts that have precedent in the previous literature, our study provides the first detailed within‐study comparison of these common MID task contrasts. The findings demonstrate similarity between positively and negatively arousing anticipation cues, apparent deactivation of striatal regions during the outcome phase, dissimilarity between Big Win > Small Win anticipation and other anticipatory contrasts, and relatively weak associations between MID task activations and self‐reported behaviors. These findings are generally consistent with previously reported MID task‐specific conceptual findings (Bjork et al., 2010; Oldham et al., 2018) and also have implications for task‐general theoretical problems (Hedge et al., 2018; Price & Friston, 1997).

A relatively similar pattern of group‐level activation was observed during the Big Win anticipation and the Big Loss anticipation phase. A direct comparison of Big Win versus Neutral and Big Loss versus Neutral anticipation contrasts revealed negligible differences between the activation in the VS and insula in the group‐level activation maps, and only a small Win‐related increase in activation in the mPFC. This similarity in activation profiles during anticipation of both positive and negative stimuli is consistent with a recent meta‐analysis demonstrating that approach and avoidance behavior have considerable overlap in activation (Oldham et al., 2018), and other studies reporting similar activation patterns in young adults (Joseph et al., 2016; Murray et al., 2020) and populations at risk to substance use (Bjork et al., 2008). The similarity in the neural activation to the anticipation of Big Win and Big Loss cues is also consistent with the hypothesis that certain regions may display roughly equivalent activation at the extreme ends of value (Bartra et al., 2013). This may suggest alternative cognitive processes (such as attention or motivation) that may be involved during the anticipation phase (Abler et al., 2006; Breckel et al., 2011; Krebs et al., 2012; Schouppe et al., 2014), as the VS may facilitate detection and attention to cues (Peters et al., 2011) as it serves as a limbic‐motor interface that converts signals into action (Floresco, 2015). The overlap between win and loss group‐level activation suggests the activation maps are more comparable than different which may correspond to a shared cognitive process (Price & Friston, 2005).

However, there was one notable instance in which our analysis revealed dissimilarity between contrasts during the anticipation phase. Although the Big Win versus Small Win contrast activated striatal regions, the contrast demonstrated a limited association with other contrasts in the anticipation phase. Specifically, in group‐level activation, there was much greater similarity between Big Win versus Neutral and Big Loss versus Neutral contrasts than the similarity between Big Win versus Neutral and Big Win versus Small Win contrasts. Given that the MID task activates a broad set of regions involved in effortful initiation and anticipation (Suzuki et al., 2020), subtraction of cues with lower effort and greater variability (e.g., neutral stimuli) from higher effort and lower variability (e.g., Big Win), versus with those with slightly more effort (e.g., Small Win), may change the amount of preparatory signal subtracted from the contrast map. It is likely that beyond the cognitive process of “wanting,” there are co‐occurring cognitive processes in these cues which may violate assumptions when using subtraction to infer reward sensitivities (Caplan, 2007).

Our comparison of positively and negatively valenced reward outcome contrasts revealed widespread *deactivation* throughout the brain during the outcome phase. These patterns were counter to a recent meta‐analysis, using activation likelihood estimation (based on nine studies), that reported increased activation in reward outcome (Oldham et al., 2018). Oldham et al. (2018) reported increased activation during the outcome phase in the Reward Hit versus Reward Miss or Reward Hit versus Neutral contrasts (see Table 2 in Oldham et al., pg 3404). However, our deactivation results differed from Oldham et al. (2018) in that we focused on the Reward Hit versus Neutral Hit outcome contrast. The observed deactivation of the Reward Hit versus Neutral Hit contrast during the outcome phase is likely the spillover BOLD signal from the anticipatory phase which captures the undershoot (Buxton, 2012). In direct plots of BOLD of outcome within‐condition (e.g., Big Win hit and Big Win miss signal), this undershoot is still apparent. Although comparing within‐condition outcomes, or more complicated contrasts (Bjork et al., 2011; Veroude et al., 2016), are more appropriate when modeling the outcome phase, researchers should remain cognizant that these trials are still unbalanced (e.g., more hit versus miss trials) and underpowered (anticipation trial is bifurcated during outcome). Given the undershoot, if the neural process of interest is specific to the outcome phase, designs that temporally separate the outcome phase should be considered (Bjork et al., 2010; Murray et al., 2020).

Bearing in mind that our sample is at the developmental peak of sensation seeking (Romer, 2010; Steinberg et al., 2018), a psychological characteristic that is hypothesized to be central to the motivation toward reward (Case y, 2015; Ernst & Luciana, 2015; Spear, 2011), it is worth to consider how the association between reward activation and sensation seeking changes across anticipatory contrasts. While we found a negligible association between sensation seeking and bilateral VS activation during Big Win versus Neutral contrast (*r* < −.03), Big Loss versus Neutral has a notable negative association with sensation seeking (*r* = −.09 ‐ −.10). Then, in the context of the right VS, activation during Big Win versus Small Win contrast and sensation seeking are positively associated (*r* = 0.12). These effects may in part be consistent with the hypothesis that higher sensation seekers would be more motivated by positive rewards (e.g., win) and less affected by negative rewards (e.g., loss). However, while these distinctions may be well reasoned from a neurodevelopmental perspective (Casey , 2015) and other work reporting neural associations with sensation seeking (Cservenka et al., 2013; Hawes et al., 2017; Tapia León et al., 2019), the similarity in the negative association between right VS activity and sensation seeking across the All Win versus Neutral (*r* = −.10) and Big Loss versus Neutral (*r* = −.09) makes it difficult to discern what the key distinguishing factor is in this brain‐behavior association. Although the aforementioned examples refer to the most probable *r*‐values, it is important to remember that the 95% confidence interval in all cases crossed zero and so in some samples the association may include results in the opposite observed direction, which should limit our confidence in the interpretation.

Hence, it is critical to consider how patterns of activation across task phases/conditions relate to behaviors, since the MID task is used in a broad clinical and behavioral literature. In our analysis using psychosocial and clinical criterion measures, we found limited evidence for associations with activations across different phases and conditions. Specifically, the majority of associations between neural activation during the MID task and behavior were likely to be relatively small or negligible. As the original task design focused on clinical populations (Knutson & Heinz, 2015) and reviews suggest a robust role of limbic regions in substance use (Balodis & Potenza, 2015) and psychosis (Radua et al., 2015), this may in part explain the weak effects found in our young adult community sample. Although we cannot rule out that this lack of robust associations with behavior may have been due to features of our sample or measures, it stands in stark contrast to the large array of previous studies reporting associations of MID task activations with various real‐world outcomes (Boecker et al., 2014; Büchel et al., 2017). Further, our findings are broadly consistent with recent work that has reported a distinct contrast between the effects found in studies with and without preregistration (median *r* = 0.16 versus 0.36; Schäfer & Schwarz, 2019) and with findings in large, diverse data sets which indicate that neuroimaging markers often explain only very small portions of the variance in behavioral outcomes of interest (Marek et al., 2020; Nees et al., 2012; Paulus et al., 2019; Paulus & Thompson, 2019). This has led some to suggest that small effects are the “new normal” in clinical neuroscience research (Paulus & Thompson, 2019) and that MRI studies require especially large sample sizes (>2000) to identify meaningful effects in brain‐behavior associations (Marek et al., 2020). However, this issue needs to be explored further, as some proposed sample sizes of > 160 in univariate fMRI analyses to be reasonable (Grady et al., 2020).

One reason for discrepancy between our results and prior reports of more robust MID task associations with behavior is that effect sizes may have been overestimated in previous studies with smaller samples. Some studies have reported relatively moderate to large effect sizes (*r* > 0.25) with respect to brain‐behavior associations (Cope et al., 2019; Karoly et al., 2015), but despite the numerous brain‐behavior tests performed here that focused on related behavioral constructs, our effect sizes were consistently *substantially* lower (97% out of 400 observations, *r* < 0.20). Until recently, neuroimaging studies of individual differences have frequently been underpowered (Cremers et al., 2017; Yarkoni, 2009), with a median sample size of < 50 (Szucs & Ioannidis, 2020), which tends to cause the size and replicability of effects to be dramatically overestimated due to a combination of noise in small samples and the “statistical significance filter” (Gelman & Loken, 2014; Vasishth et al., 2018). Our findings suggest that researchers should be prepared for relationships between MID task activations and clinical or real‐world outcomes of interest to be of small size and design their studies accordingly. The use of large data sets from collaborative efforts (e.g., ABCD: Casey  et al., 2018) may be preferable to smaller samples collected by individual laboratories (Beltz & Weigard, 2019; Paulus & Thompson, 2019) and would be valuable in reexamining the results presented here to understand how effects change.

Beyond the possibility that effect sizes in previous MID studies may have been inflated by small sample sizes and flexible selection of contrasts, the lack of relationships may also be attributed to problematic validity of fMRI‐based tasks and the underlying assumptions about the cognitive processes involved, such as positive or negative valence. A large proportion of tasks in fMRI are experiment based, whereby conditions are manipulated to evoke excitation of a specific cognitive process (Price & Friston, 1997). Although the MID task evokes distinct neural processes that are consistent with current conceptualizations of the mesolimbic system (Knutson & Greer, 2008), the classic metric of validity, namely that a test measures the psychological trait that it claims to measure (Cronbach & Meehl, 1955; Kelley, 1927), appears to be underexplored in the implementation of this paradigm for assessing brain‐behavior relationships. In fMRI studies of individual variation, such as behavioral differences that may be associated with neural measures of reward, the combination of experimental and correlational methods is required, work that arises from two distinct traditions in psychology (Cronbach, 1957). Correlation research attempts to increase between‐individual variation, whereas experimental work attempts to limit or control for the between‐individual variation; the latter methodological approach practice has been argued to contribute to poor predictive effect of cognitive measures in correlational research (Dang et al., 2020). Together, the weak predictive effect of select cognitive tasks and poor test–retest of univariate fMRI (Elliott et al., 2020) can contribute to the unreliable estimates of different task contrasts and the interchangeable use of contrasts will inevitably result in playing “20 questions with nature” (Newell, 1973).

The inferential processes in task‐based fMRI pose conceptual challenges. It has been argued that the standard approaches in task‐based fMRI that utilize the technique of subtracting conditions are fundamentally flawed in achieving the isolation of the neural substrates of specific mental functions (for discussion, see: Cacioppo et al., 2003; Caplan, 2007; Price & Friston, 2005). Poldrack and Yarkoni (2016) suggest that there are basic conceptual difficulties within subtraction applied in task‐based fMRI “that remain widely underappreciated within the neuroimaging community” (pg. 589). This is observed in the MID task, as *conceptually* the subtraction intends to measure approach and avoidance of positive and negative conditions (Knutson & Greer, 2008), but this is not consistent in the activation patterns of valence (insula) and approach (VS/Nucleus Accumbens) structures that, at the group level, are activated similarly in both conditions (Murray et al., 2020; Oldham et al., 2018). Although using monetary value allows control of magnitude, probability, and timing (Knutson & Greer, 2008), adding a discrete step with positive or negative monetary cues (i.e., “pure insertion assumption”; Price & Friston, 1997) may not be sophisticated enough to identify valence and approach over and above processes of attention and/or motivation within an individual. While the MID task measures distinct positive and negative valenced systems in two distinct phases, the nature to which these phenomena vary or are consistent across specific behaviors has not been well characterized. And in fact, our work in a community sample of young adults suggests that they may not significantly differ in terms of the structures that are involved.

Although our findings suggest a high level of variability between contrast choices and behavioral associations, several measures can be taken that may improve the generalizability of results in the MID task literature. First, an immediate step that can be taken by researchers is increasing sample sizes in task‐based fMRI research. Currently, a large proportion of fMRI studies are substantially underpowered for finding the effect they are testing (Szucs & Ioannidis, 2017, 2020). Second, researchers would benefit from assessing how the MID contrast values fit in a larger nomological network of neural and behavioral constructs (Poldrack & Yarkoni, 2016), beyond an abstract subtraction processes that presume a process of motivation or consumption of reward and preregister these hypotheses in advanced. Third, multivariate methods, such as dimensionality reduction and cross‐validated predictive modeling, may help with the reproducibility of theorized neural substrates of cognitive processes (Hong et al., 2019). Multivariate, cross‐validated analyses can provide a priori activation patterns and locations that can be confirmed out of sample, reducing the possibility of exploring multiple hypotheses. Finally, if the goal is to characterize individual variability in neural function, researchers should implement functional organization techniques to explain changes in behavior and cognitive processes (Beltz et al., 2016; Yip et al., 2019; Zhang et al., 2019). Network models of task‐based fMRI may be particularly helpful for uncovering the neural architecture of cognitive processes (Greene et al., 2018; Medaglia et al., 2015). By using individual‐ and group‐level estimates of connectivity patterns (Beltz et al., 2016), task‐based analyses may improve the identification and replication of neural signatures that will aid researchers studying developmental and clinical differences (Yip et al., 2019; Zhang et al., 2019).

### 5.1 Limitations

Although the findings here pose significant implications, there are multiple limitations. First, the nature of our findings are tested only in a modified version of MID task that was administered in a young adult sample, so the implications should be considered and confirmed in a separate sample(s) to determine which effects converge between samples and which are limited to a sample. Future work should examine these associations in a larger sample and at different developmental stages using the ABCD study data. Second, the correlates between ROI activation and self‐reported behavior may be underestimated, such that behavior that is collected contemporaneously with the scan acquisition or in the nature that the brain predicts behavior may produce different effects. Moreover, due to a combination of increased number of voxels and alternative methods for controlling the false positive rate, the whole brain statistical analyses exploring brain‐behavior associations may reveal findings that an ROI constrained analysis may overlook. Third, only a subset of common a priori contrasts were selected from the literature. Alternative contrasts, such as the linear combination of winning or alternative contrasts during the outcome phase, should be considered in future work. Since the anticipation and outcome phase in this task were not jittered, we could not directly contrast these phases at the individual level (only group level), due to risk of collinearity. Finally, due to the outcome phase containing variable number of trials as a function of 60% accuracy rate, the activation patterns may be influenced by the surprise of the event(s) (Vassena et al., 2020), which should be considered in future work.

It is worth noting that some of the differences between positive and negative cues in our and previous studies may depend on age‐related factors and sample characteristics. For instance, while our results did not demonstrate a meaningful difference in the activation of the VS or insula between Big Win and Big Lose anticipation phases, age‐related differences have been previously reported using this task, such that increases in activation during Big Win anticipation trials were greater in older adults (Bjork et al., 2010), and reduced activation in response to Big Lose anticipation in 9‐ to 12‐year‐olds (Cope et al., 2019). This suggests patterns of activation during the MID task within and between sample comparisons has been considered when age‐related effects are present, as qualitative differences between some contrasts may not be easily apparent. Furthermore, whereas these analyses focus on a community‐recruited young adult sample, previous reviews focused on clinical population (Balodis & Potenza, 2015; Radua et al., 2015), and these results should be considered in the future within a clinical population to assess how associations would change in light of clinical factors.

## 6 CONCLUSION

Although univariate fMRI contrasts from the MID task are often used to measure neural substrates of reward processing, modeling techniques have varied substantially between studies. The structure of the task has been proposed to separately measure the constructs of arousal and valence. However, it is still unclear whether these dimensions are easily separable using different task contrasts, and whether findings from different contrasts can be easily generalized between studies. Our within‐sample comparison of MID contrasts during multiband fMRI revealed more similarities than differences between positive and negative cues during the anticipation contrast, dissimilarity of the specific Big Win versus Small Win contrast during the anticipation phase, a robust deactivation effect in the outcome phase, and behavioral associations that are less robust than previously thought. These findings point to the need for caution in future work that make attempts at generalization and encourage researchers to power their studies for effects that may be smaller than previously hypothesized.

## CONFLICT OF INTEREST

The authors declare that they have no conflicts of interest.

## AUTHOR'S CONTRIBUTION

MD conceived the study; MD, KG, and AW conducted the statistical analysis; MD wrote the initial draft of the manuscript, and KG, AW, HJ, and AJ provided support with the analyses, drafts of analyses, and results. DK and EH designed and executed the survey and the neuroimaging protocol. KG, AW, HJ, AJ, EH, and DK assisted MD with study writing and revisions. All authors read and approved the manuscript.

### PEER REVIEW

The peer review history for this article is available at https://publons.com/publon/10.1002/brb3.2093.

## Supporting information

Supplementary Material

## Data Availability

Readers seeking access to this data should contact the lead author Michael Demidenko (demidenm@umich.edu) or senior author Dr. Daniel Keating (keatingd@umich.edu). Access will be granted to named individuals in accordance with ethical procedures governing the reuse of sensitive data. Infrastructure is currently being developed in collaboration with the Inter‐university Consortium for Political and Social Research (ICPSR) at the University of Michigan (https://www.icpsr.umich.edu) to archive and share data in an ethically approved manner and will be shared at a later TBD date.
