# Supplementary material for: Interactions between methodological and interindividual variability: How Monetary Incentive Delay (MID) task contrast maps vary and impact associations with behavior
Source: Brain Behav. 2021 Mar 22;11(5):e02093. doi: 10.1002/brb3.2093 (PMC8119872; doi:10.1002/brb3.2093)
Supplement: Supplementary file 1 — Supplementary Material [file BRB3-11-e02093-s001.docx]

1. **fMRI Analyses**
   1. *Task Paradigm:*

Each cue lasts for 2000ms and is followed by a jittered fixation cross (1500-4000ms). Following the jittered fixation cross, the target probe cue (187-500ms) appears that requires participants to respond in order to win or not lose money. If participants are too fast (or too slow), that trial is marked as incorrect. Following the probe phase, the outcome phase (2000ms minus the target duration) indicates the outcome of that trial (for example, ‘You Win $5!’, ‘You Keep $0.20’, ‘You Lose $5’ or ‘No Money At Stake!’). The task is individualized with an initial mean response time (MRT) that is used from the practice run performed outside of the scanner minutes before the scan. Using the average reaction time (RT) plus two standard deviations on correct trials, the MID task individualizes the difficulty to reach around 60% accuracy rate by adjusting the difficulty (that is, probe duration). Prior to the scan, participants were informed of all cue-related outcomes and completed a practice trial of the MID task. Participants were explicitly told that their performance on the task during the scan (for example, $5 Win Cue is was associated with an opportunity to win $5 and a $5 Lose cue was associated with an opportunity to not lose $5) will be associated with the compensation they can get for their cumulative earnings during the MID (Maximum $30). Stimuli were presented via the IFIS system (MRI Devices, Inc., Milwaukee, WI), an integrated stimulus display and experimental control package, also capable of recording button presses. Lenses were available with the IFIS system to correct subject vision, as needed.


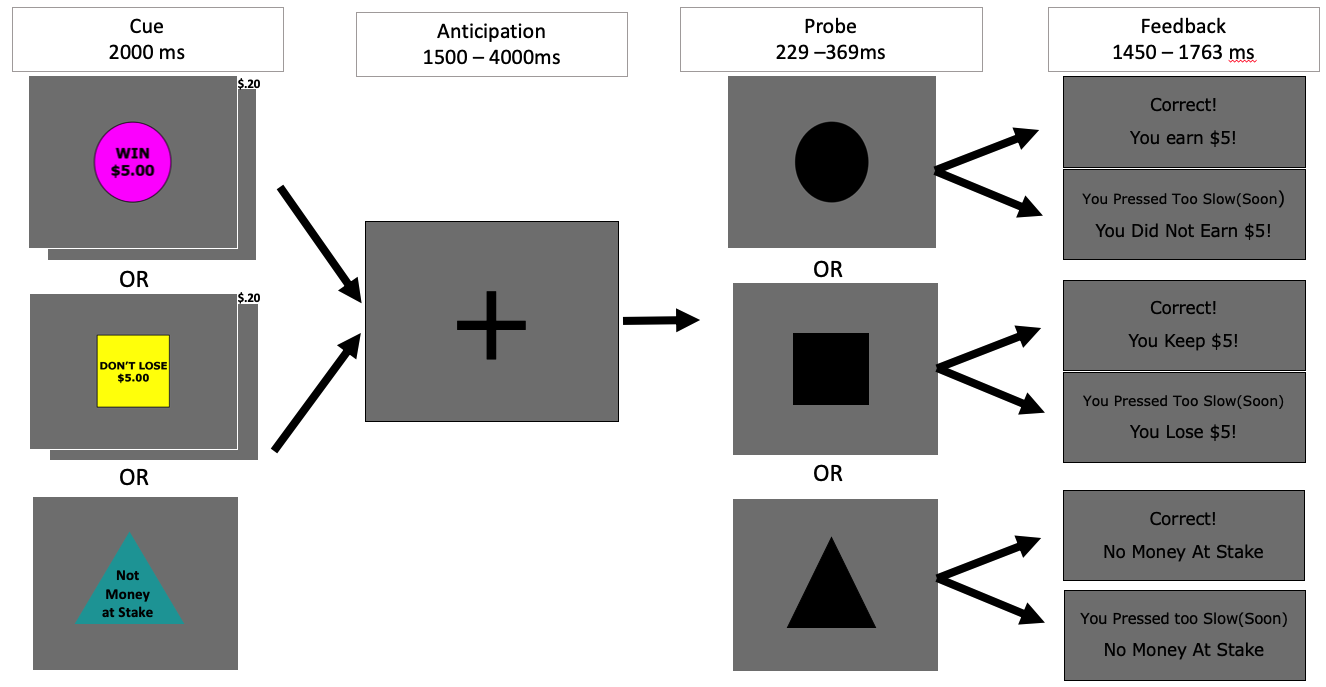


- 1. *Motion:*

There were no subjects with motion estimates that exceeded the mean FD > .9. Estimates were derived using the fsl_motion_outliers command applied to both A and B runs. Motion is reported for A) diacom reconstructed runs (preFD), averaged across both runs, and B) preprocessed (physio-, motion-, and fieldmap corrected filtered_func files) runs (postFD), averaged across both runs.

|  | Mean (FD) | Min (FD) | Max (FD) |
| --- | --- | --- | --- |
| PreFD | .11 | .03 | .36 |
| PostFD | .02 | .01 | .08 |

- 1. *Modeled Contrasts and Design Matrix*

Ten distinct contrasts were modeled for distinct EV’s in the GLM model (A-U). Each contrast, is described below, including an example FSL Feat GLM matrix, excluding the motion parameters. Each EV from the MID task was included in the Feat design using a 3-column format. Anticipation events included: Big Win (BW), Small Win (SW), Neutral (N), Small Lose (SL), and Big Lose (BL) condition cues, as well as the modulator of Expected Value of BW and Expected SM. Outcome included both hit and miss events for: BW, SM, N, SL, BL, and parametric modulator of Positive Prediction Error (BW & SW Hit) and Negative Prediction Error (BW & SW Miss).

1. Anticipation Win vs Neutral
2. Anticipation Big Win vs Neutral
3. Anticipation Big Win vs Small Win
4. Anticipation Big Win vs Implicit Baseline (e.g. mean signal)
5. Anticipation Big Lose vs Neutral
6. Outcome Big Win Hit vs Neutral Hit
7. Outcome Lose Big Hit vs Neutral Hit
8. Win [$5 + $0.20] Expected Value
9. Positive Prediction Error [$5 + $0.20 Reward Hit]
10. Negative Prediction Error [$5 + $0.20 Reward Miss]

Below are the parameters modeled in the design matrix in FSL feat first level (not shown in the figure are the motion parameters). Estimates include the anticipation, outcome and prediction error phase.

1. Big Win [Anticipation]
2. Small Win [Anticipation]
3. Neutral [Anticipation]
4. Small Lose [Anticipation]
5. Big Lose [Anticipation]
6. Big Win Hit [Outcome]
7. Big Win Miss [Outcome]
8. Small Win Hit [Outcome]
9. Small Win Miss [Outcome]
10. Neutral Hit [Outcome]
11. Neutral Miss [Outcome]
12. Small Lose Hit [Outcome]
13. Small Lose Miss [Outcome]
14. Big Lose Hit [Outcome]
15. Big Lose Miss [Outcome]
16. Expected Value: Big Win
17. Expect Value: Small Win
18. Positive Prediction Error: Big Win Hit
19. Positive Prediction Error: Small Win Hit
20. Negative prediction Error: Big Win Miss
21. Negative Prediction Error: Small Win Miss


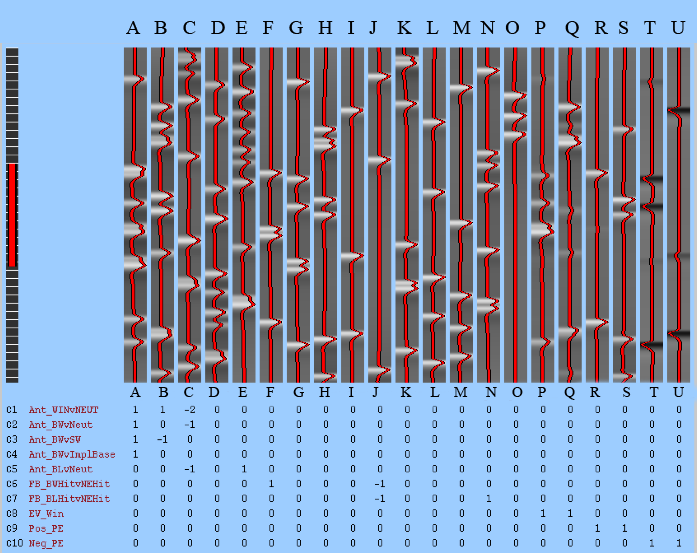


- 1. *Regions of interest: Below are the MNI coordinates (Table S1) for regions used in theses analyses, and a representation of regions on a glass brain representing the location (Figure S1).*

**Table S1**. *A prior* MNI coordinates pulled from Neurosynth

| Region of Interest | Index | Glass Brain Index | MNI Coordinate  (x, y, z) | | |
| --- | --- | --- | --- | --- | --- |
| Right Ventral Striatum | R_VS | Blue | 15 | 8 | -9 |
| Left Ventral Striatum | L_VS |  | -12 | 8 | -8 |
| Medial Prefrontal Cortex | mPFC | Green | 2 | 40 | -8 |
| Anterior Cingulate Cortex | ACC | Pink | 3 | 29 | 21 |
| Left Orbitofrontal Cortex | L_OFC | Yellow | -22 | 34 | -14 |
| Right Orbitofrontal Cortex | R_OFC |  | 32 | 33 | -14 |
| Right Insula | R_Insula | Red | 38 | 13 | -4 |
| Left Insula | L_Insula |  | -38 | 12 | -9 |


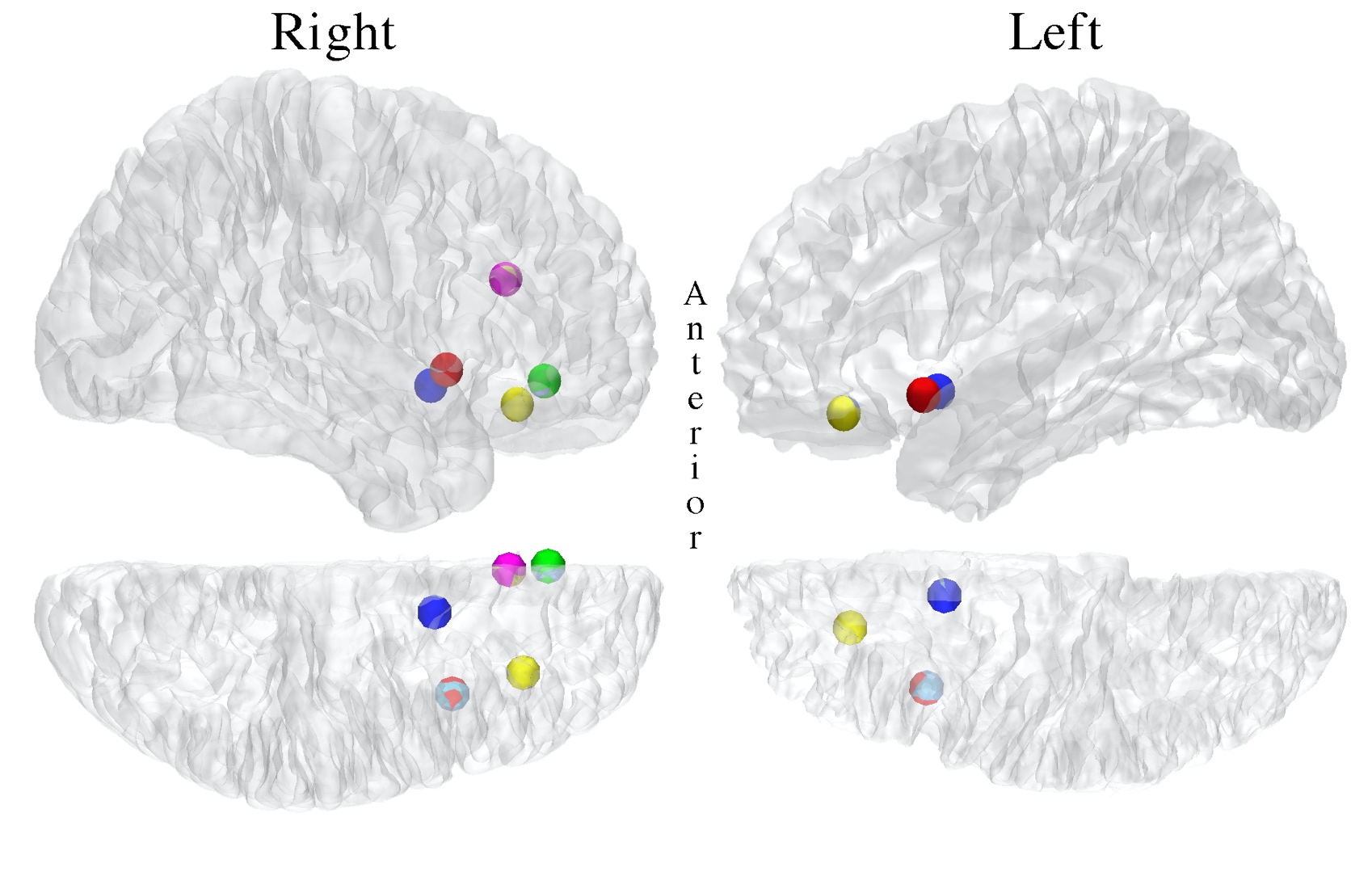


***Figure S1***: MNI Glass Brain Representing Location of Regions of Interest used in Analyses

Note: Index of colors in available in Table S1; Lateral and Axial Views of Left & Right Hemisphere

**Table S2.** Paradigm Contrast Reviews PubMed 2015-2019

| Study | N | age ranges | Paper Focus | Target % Acc | Type of Contrast (Anticipation = Ant; Outcome = Out) | MID version |
| --- | --- | --- | --- | --- | --- | --- |
| (Dhingra et al., 2019) | 54 | 22-74 | Age related effect of reward activation | 67% | **Ant:** Big Win v. Neutral,  Big Win vs Small Win,  Small Win vs Neutral | modified MID |
| (Schwartz et al., 2019) | 15 | 15.29 (2.4) | Social vs non-social reward | 66% | **Ant** : Reward vs No Reward | Youth friendly - MID - Pinata task (non-social) |
| (Swartz et al., 2019) | 262 | 16.8 (.58) | Mexican origin, alcohol | - | **Ant:** Reward v. Neutral  **Out**: Reward Hit v. Neutral hit | MID |
| (Maresh et al., 2019) | 99 | 13-19 | EEG-Bipolar Disorder | 70% | Average each trial | MID |
| (Nestor et al., 2019) | 36 | 16.2 (most male) | cannabis dependence | 50% | **Ant**: Win v. Neutral | MID |
| (Aloi et al., 2019) | 150 | 16.1 (1.1) | Diff. in alcohol and marij. Exposure | 66% | ***Modulator: reward value outcome and win vs loss*** | Altered version, trails: Neut 12; Win 48; Loss 48 |
| (Cope et al., 2019) | 34 children (10.5) | 16 | substance use -- eternalizing and FH | 60% | **Ant:** Big Win v. Neutral  Large Loss v. Neutral | MID - modified Mich. Long. Study |
| (Cao et al., 2019) | 1510 | 14yr | sensation seeking, reward anticipation. [Reward = candy] | 66% | **Ant**: Big reward v. Neutral  **Out**: Big Reward Hit v. No Reward; Big Reward Hit > Neut Reward Hit | MID [IMAGEN] -- No loss cue |
| (Stevens et al., 2018) | 151 | 12-18 | ADHD | 66% | **Ant**: Big Win v. Small Win | Modified Knutson ($0, $1, $5) |
| (Papanastasiou et al., 2018) | 298 | 14 & 19 | psychotic like experiences |  | **Ant**: Big Win v. Neutral | Knutson? Not specified…. |
| (Landes et al., 2018) | 54 | 12-17 | major depressive disorder-ERP/EEG | 50% | Average each trial | altered MID |
| (Martz et al., 2018) | 57 | ~20 | psychosocial/neural resilience in FH SU | 60% | **Ant**: Win v. Neutral | MID - modified Mich. Long. Study |
| (Chronaki et al., 2017) | 32 | 10-16 | Reinforcement effects | 66% | Average each trial | modified versoin, shapes, colors. |
| (Navas et al., 2018) | 68 | 16.5 (1.5) | Body fat | NR | **Anticipation** linear: big>medium>low>neutral.  **Out**: Win Hit vs Win Miss | modified colors, shapes, etc. |
| (Garrison et al., 2017) | 14 & 28 | 17 (1.3) | Smoking Behavior | 66% | [**Ant:** Win v. Implicit Baseline  Loss v. Implicit Baseline](https://reader.elsevier.com/reader/sd/pii/S0006322314006520?token=9A0DEB5A34F300DF7F5E144E1C1E05C7E5518FE9A88033F71E11280C14B0F7D36C9E6B78249E4C571166AC175247529B) | Modified Knutson MID -- no paradigm example |
| (Colich et al., 2017) | 76 participants (38 pairs) | 9-15 | Risk for depression | 75% | **Ant:** Win v. Neutral  Loss v. Neutral | modified - Kid MID task (replaced money w/ points of Knutson 2008 version |
| (Xu et al., 2017) | 1129 | 14.4 (.4) | ADHD | 66% | **Ant**: Win v. Neutral | MID [IMAGEN] -- No loss cue |
| (Bourque et al., 2017) | 246 & 1196 | 14.3 (.4) - | Symptoms at 16 - Psychotic like experi. | 66% | **Ant**: Win v. Neutral | MID [IMAGEN] -- No loss cue |
| (Duka et al., 2017) | 1299 | 14.4 (.4) | gene & brain response correlates | 66% | **Ant**: Big Win v. Neutral | MID [IMAGEN] -- No loss cue |
| (Gonzalez et al., 2016) | 83 | ~25 | Neighborhood quality on brain | 66% | **Ant:** Win v. Neutral | MID (27 reward, 27 punish, 18 neutral cues) |
| (Büchel et al., 2017) | 144 | 14 (16 f/u) | Novelty seeking and substace use | 66% | **Ant**: Big Win v. Small Win | MID [IMAGEN] -- No loss cue |
| (Veroude et al., 2016) | 328 | 17.6 (3.3) | Callous-unemotional, ADHD, oppositional defiant | 33% | **Ant**: Win v. Neutral **Out** {reward hit.- reward miss} > {non-reward hit-non_reward miss} | MID modified - colored cues (green/red), NO LOSS condition |
| (Mikita et al., 2016) | 1472 | 14.4 | Autism | 66% | **Ant**: Big Win v. Neutral **Out**: Big Win Miss v. Miss Neutral;  Big Win Hit v. Neutral Hit | MID [IMAGEN] -- No loss cue |
| (Urošević et al., 2016) | 47 | 13 - 19 | bipolar disorder | 70% | **Ant**: gain vs neutral; large gain > small [compensated $ for 10% of winnings] | MID - 60 trials |
| (Richards et al., 2016) | 429 |  | Plasticity genes & social environments | 33% | **Ant**: Win v. No Reward.  **Out**: Reward Hit v. Reward Miss | Red = no reward squared; green = reward screen (no loss condition) |
| (Joseph et al., 2016) | 27 & 51 (78 total) | 11-14 & 18-25 | personality (impulsivity, avoidance, approach tendencies) | 66% | Contrasts are the demeaned versions of contrasts [1, 0.1, 0, .1, 1] and [− 1, 0.1, 0, 0.1, − 1] | Modified MID |
| (Martz et al., 2016) | 108 | 21-23 (3 fMRI scans) | Parental History SUD | 66%% | **Ant**: Big Win v. Small Win | modified MID - MLS |
| (van Hulst et al., 2015) | 18 | 9.5-14.5 | Pilot Child Friendly MID | NR | **Ant**: Big Win v. Smal Win **Big Win v. Neutral** | Child Friendly MID |
| (Mori et al., 2016) | 30 | 18-19 | Depression | NR | **Ant:** Big Win v. No Gain,  Large Loss v. No Loss | MID (40 gain, 40 loss, 10 neutral trils) |
| (Li et al., 2015) | 26 | 18.7 (1.4) | DCM of MID | 66% | **Ant:** Win vs Neutral  Loss v. Neautral, **all vs neutral** **used in DCM)** | Modified MID 60 total trials (20, 20, 20) |
| (Chan et al., 2016) | 28 | 18.9 (1.8) | Social & Monetary Process, Anhedonia | 66% | **Ant:** Win v. Neutral,  Lose v. Neutral.  **Out**: Win Hit v. Neutral Hit Loss miss v. Neutral Miss. | Modified MID 60 total trials (20, 20, 20) |
| (Karoly et al., 2015) | 138 | 14-18 | Substance Use | 66% | **Ant:** Win v. Neutral Lose v. Neutral | [modified MID from Filbey et al. 2013](https://journals.plos.org/plosone/article?id=10.1371/journal.pone.0061470) |
| (Sauder et al., 2016) | 38 | 13-19 | Self-injury | NR | **Ant**: Reward v. Neutral - factorial design of reward magnitude x group. | MID |
| (von Rhein et al., 2015) | 350 | 17.8 | ADHD | 33% | **Ant:** Win v. Neutral  **Out**: Win Hit v. Neutral Hit | modified MID 25 reward and 25 neutral experimental trials |
| (LeMoult et al., 2015) | 38 (21/17) | 11.2 (1.2) & 13.3 (1.4) | Menarche on diurnal cortisol production | 75% | **Ant**: Gain -- unclear | KID MID task- 100 trials |
| (Nees et al., 2015) | 530 | W1. 14.3 & W2. 16.2 | BDNF and reward in early substance use | 66% | **Ant & Out w/ subj specific regressors** | MID - IMAGEN study |
| (Boecker et al., 2014) | 162 | 24/4 | Early life adversity | NR | **Ant:** Win v. No Win | mod. MID |

*Note*. Median sample size (*N* = 91; min: 15 max: 1510).

Across 37 studies 61 modeled contrasts:

*Anticipation* (n = 43, 70%):

49% - All Win > Neutral

16% Big Win > Neutral

14% Big Win > Small Win

12% Loss > Neutral

9% others (Big Loss > Neutral; Small Win > Neutral; Win (or Loss) > Implicit Baseline)

*Outcome* (n = 8, 13%):

34% Reward Hit > Neutral Hit

25% Big Win Hit > Big Win Miss

38% others (Big Win Hit > Neutral Hit; Big Win Miss > Neutral Miss; Loss Miss > Neutral Miss)

*Others* (n = 10, 16%):

Demeaned contrasts, complex outcome contrast, linear model of anticipation cues, average of each trial, and other modulators.

1. ***Results***
   1. *Demographic for Full Sample*

**Table S3**.

|  | Total  *n* = 104 |
| --- | --- |
| Sex, Female *n* (%) | 59 (56.7) |
| Race, *n* (%) |  |
| Black, non-Hispanic | 15 (14.4) |
| White, non-Hispanic | 74 (71.2) |
| Other | 6 (5.7) |
| Hispanic/Latinx | 9 (8.7) |
|  | *M (SD)* |
| Age | 19.3 (1.3) |

- 1. *MID Condition Accuracy*

Accuracy was within the range performance was expected, whereby the average performance was near 60%. The distribution was in the manner we expected, whereby Big Win and Big Lose conditions both had higher performance that small value and neutral conditions

***Table S4.*** Mean and standard deviation of accuracy

| Condition | Mean | SD |
| --- | --- | --- |
| *Overall* | *0.57* | *0.04* |
| Big Win | 0.62 | 0.10 |
| Small Win | 0.57 | 0.10 |
| Neutral | 0.48 | 0.14 |
| Small Lose | 0.57 | 0.09 |
| Big Lose | 0.60 | 0.10 |


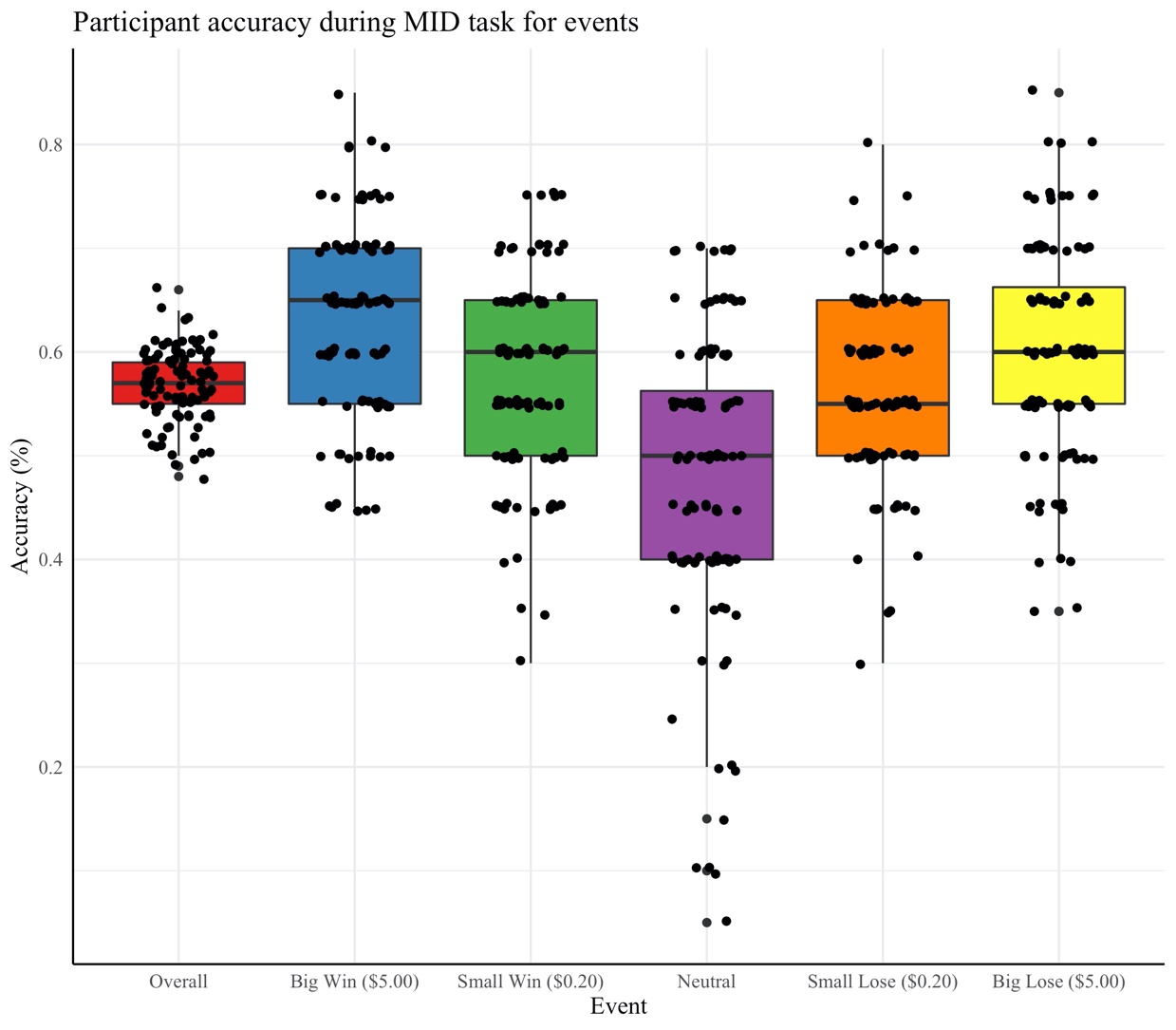


***Figure S2.*** Distribution of Accuracy by Condition Type

- 1. *Correlations between Psychological Characteristics: Correlations among aggregated self-reported items (Wave 1 – Wave 3). BSSS = Brief Sensation Seeking Scale; BIS = Barratt Impulsivity Scale – Brief*

**Table S5**. Correlations Between self-reported items (**z-scored**)

|  | 1 | 2 | 3 | 4 | 5 |
| --- | --- | --- | --- | --- | --- |
| 1. Internalizing | - |  |  |  |  |
| 2. Externalizing | .51 | - |  |  |  |
| 3. Substance Use | .04 | .51 | - |  |  |
| 4. BIS | .30 | .57 | .23 | - |  |
| 5. BSSS | .09 | .46 | .36 | .44 | - |

- 1. *Signal-to-Noise Ratio for mPFC and bilateral VS*

SNR is calculated using the 3dTstat command below. mPFC here is the mPFC peak cluster from Neurosynth for “ventromedial prefrontal” search term. For references, the **nonbrain** of SNR table is for the ROI created in a non-brain region (will contain some smoothed signal) as our filter_func_data had bet extracted, so it was most reasonable for comparison. The signal to noise is calculated by run (two runs)

$*3dTstat -prefix <output_snr_file> -cvarinv <filter_func_data inputfile>*


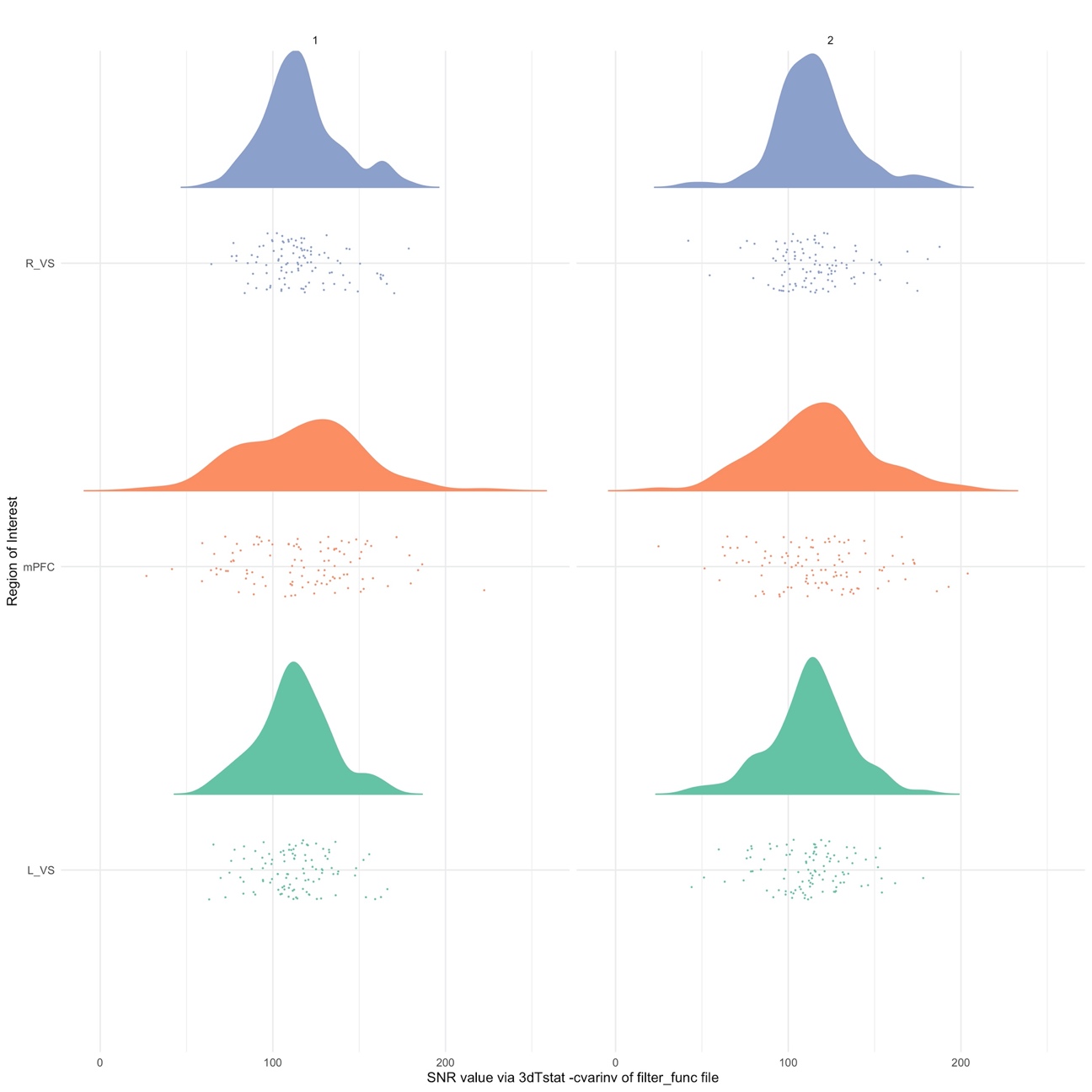


***Figure S3***: Comparison of signal-to-noise ratio for subcortical (VS) and cortical regions (mPFC)

R_VS = Right Ventral Striatum; L_VS = Left Ventral Striatum; mPFC = medial Prefrontal cortex

- 1. *Similarity Matrices*

The percent overlap between any two activation maps is defined from a set theoretical point of view, where the overlap $J(A,B)$ is defined by the well-known relation as:

$$J\left( A,B \right)=\frac{A\cap B}{A+B-A\cap B}$$

The relation calculates the ratio of ***common pixels*** that are activated across two activation maps, to the total number of pixels present in the two maps. For example, the Anticipation Win > Neutral and Anticipation Big Win > Neutral have 38% of pixels that overlap in their thresholded statistical activation group level maps (see Table 1 in manuscript for description of contrasts).


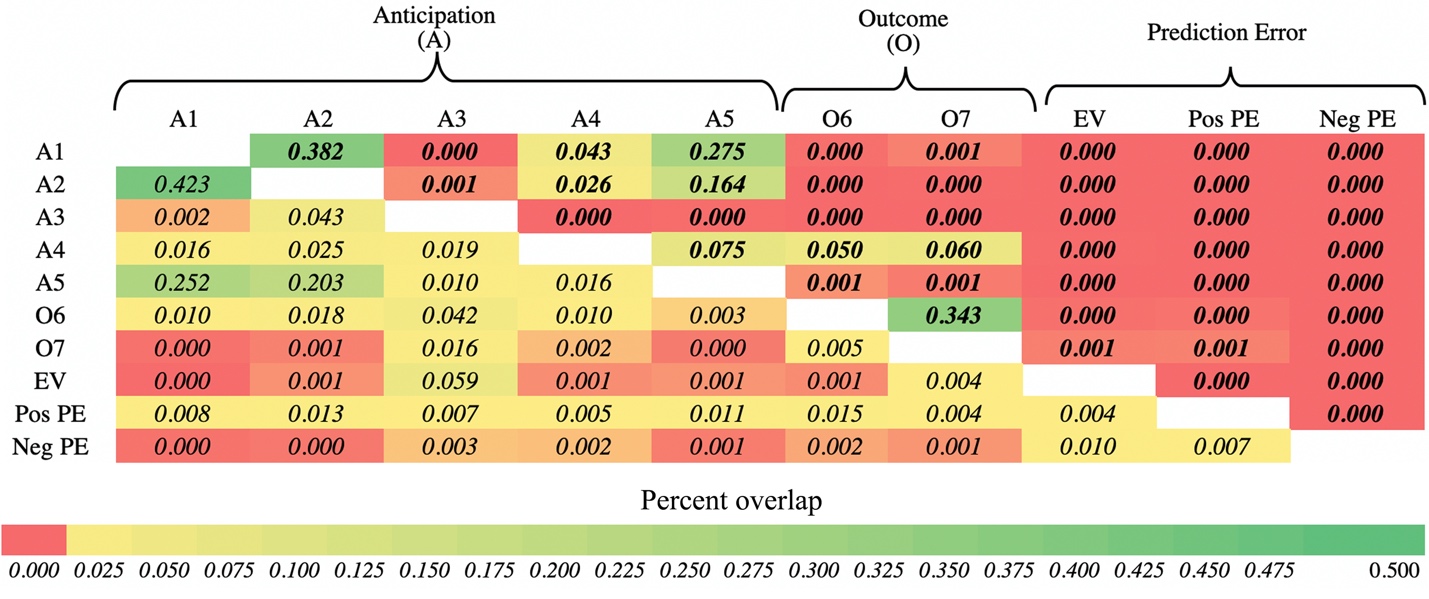


Figure S4: Similarity Matrix for thresholded individual and Group Level Maps

Note: Bolded values are group level similarity values and italicized are individual level similarities values from second level analysis.

*2.7 Brain-behavior Estimates Across complete set of contrasts (10), regions (8) and behaviors (5)*

*
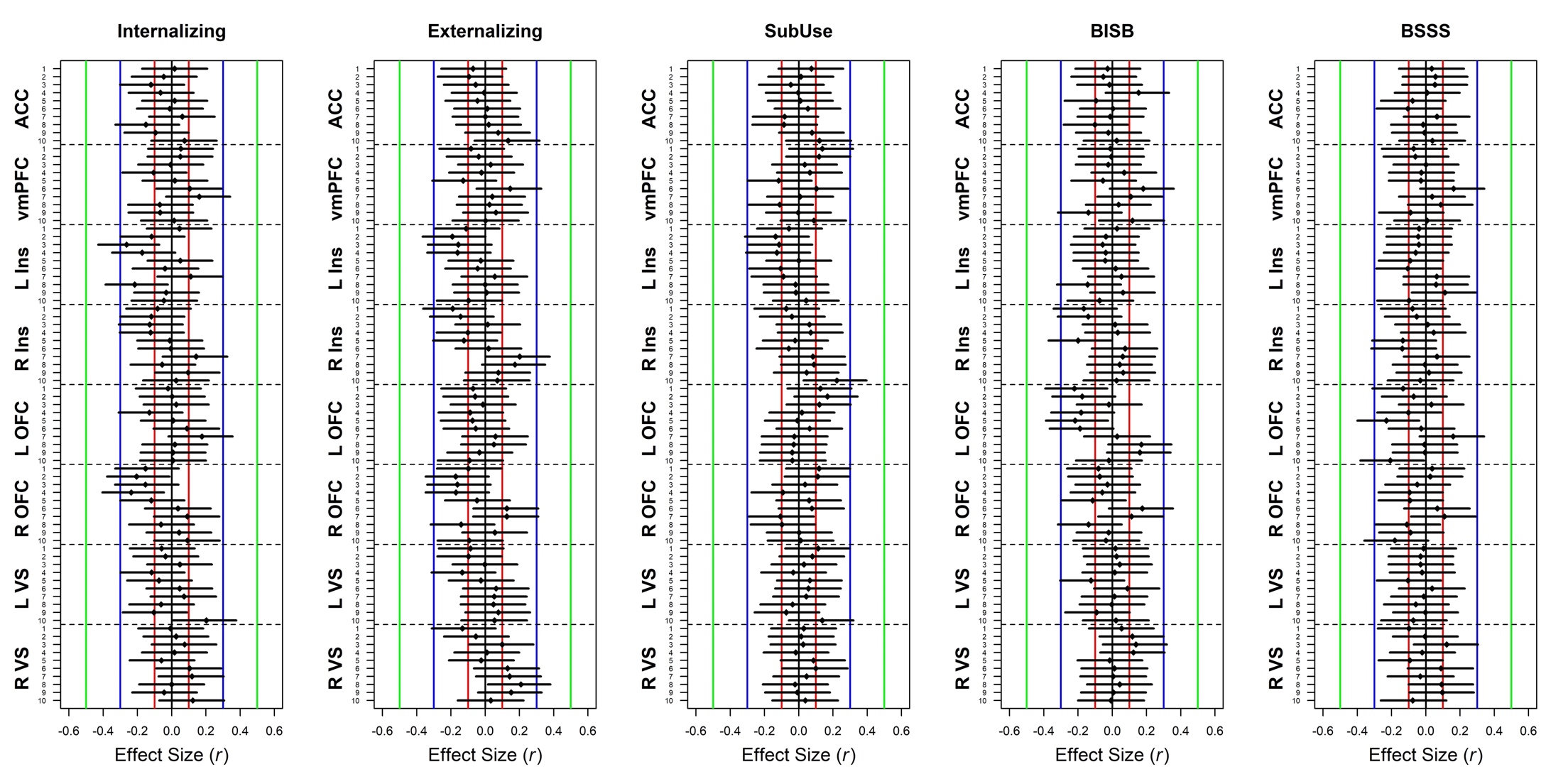
*

**Figure S5:** Forest plots displaying the most likely Pearson’s r value (black diamonds) and 95% Bayesian credible interval (black lines) for correlational relationships between ROI activation estimates from each contrast and behavioral criterion measures. Red, blue and green lines denote “small” (r=.10), “moderate” (r=.30) and “large” (r=.50) effect sizes. 1-10 = Ten contrasts listed in manuscript Table 1;

ACC = anterior cingulate cortex; mPFC = medial prefrontal cortex; Ins = insula; OFC = orbitofrontal cortex; VS = ventral striatum; L = left; R = right; SubUse = substance use composite measure; BISB = Barratt Impulsiveness Scale-Brief; BSSS = Brief Sensation Seeking Scale

*2.8 Direct observation of the BOLD signal extracted for each cortical (mPFC) and subcortical (VS) regions.*

Several steps were taken to plot the peak BOLD signal for Anticipation/outcome onset in the VS and mPFC. First, the mean signal was calculated of the preprocessed functional timeseries (t) using the calculation:

$${\% Signal Change}_{t}= \frac{t - t_{\mu}}{t * 100}$$

using fslmaths (*fslmaths* <filtered_func_data> -sub <mean_func> -div <mean_func> -mul 100 <output_name>) for each run, extracting the timeseries percent signal change for the mPFC (10mm sphere) and bilateral VS (10mm sphere). Second, the behavioral onset files (100 trials) were expanded to match their occurrence in the 651.2 second length of the task across two runs (407 volumes per run = 814 total volumes. 814 * .8sec TR = 651.2 sec). Then, the TR matched behavioral files were merged with the extracted mean percent signal changed for each ROI, and from each locked onset, 15 subsequent TRs were extracted to reflect a BOLD signal change across 12 seconds (reflecting the delayed response). For each cue onset and subsequent TR, the ***mean* and *90% Confidence Interval*** was bootstrapped to get a robust estimate of the signal change across 104 subjects for phase (anticipation or outcome) and condition type (Big Win, Big Loss or Neutral).

First we present the anticipation phase compare Big Win (LgPun) and Big Loss (LgReward) conditions. Which demonstrate small differences between valence, Win and Loss cues.

**Figure S6:** Anticipation Phase BOLD Signal change across 15 TRs.

**LgPun** = $5 Loss Cue; **LgReward** = $5 Win Cue; Error bars represent 90% Confidence Interval; p < .05 * ; p < .01**, p < .001***


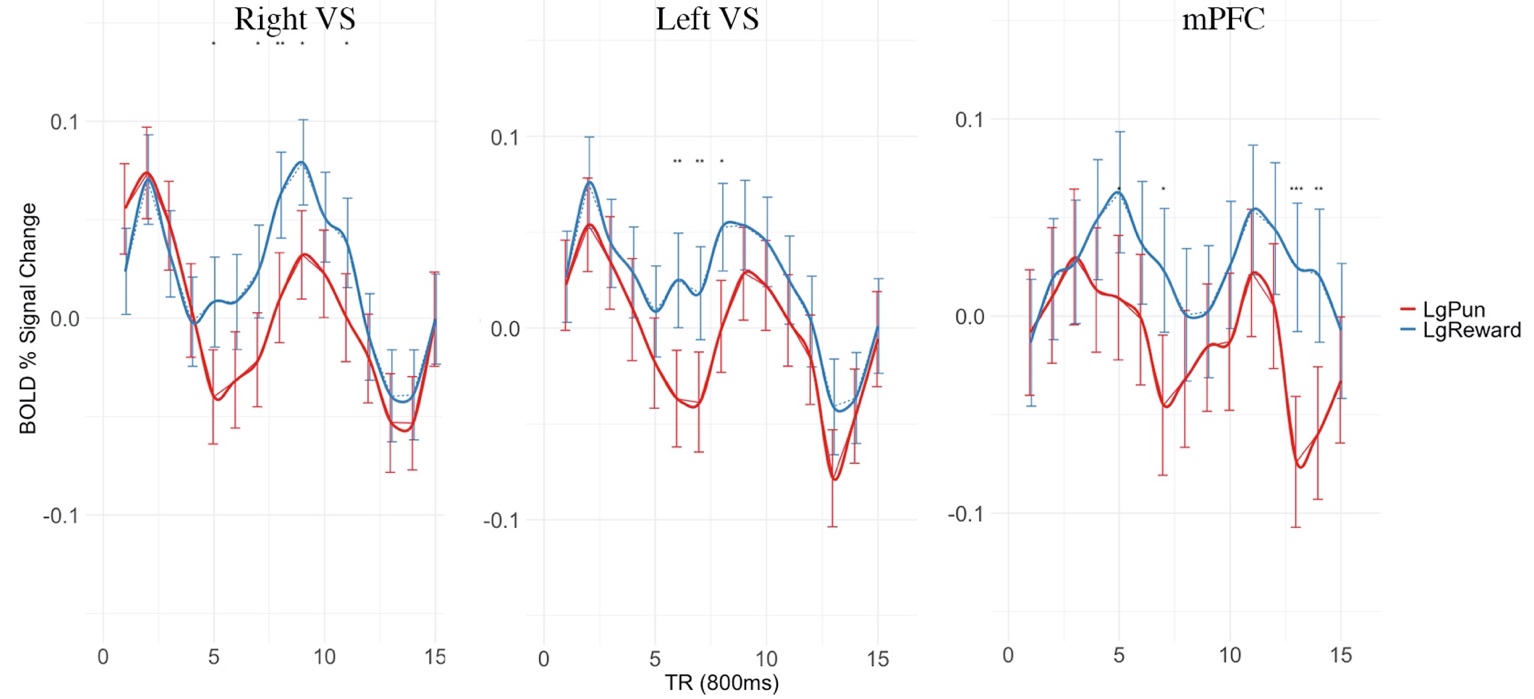


Next, we plot the signal with respect to the Outcome phase. We examine this at multiple levels: comparing Gain Hit Versus Neutral Hit (Figure S7); gain/loss Hit versus Miss (Figure S8); and general signal change for each Anticipation cue type to observe how much of the BOLD signal from the anticipation cue bleeds into the Outcome phase (Figure S9).


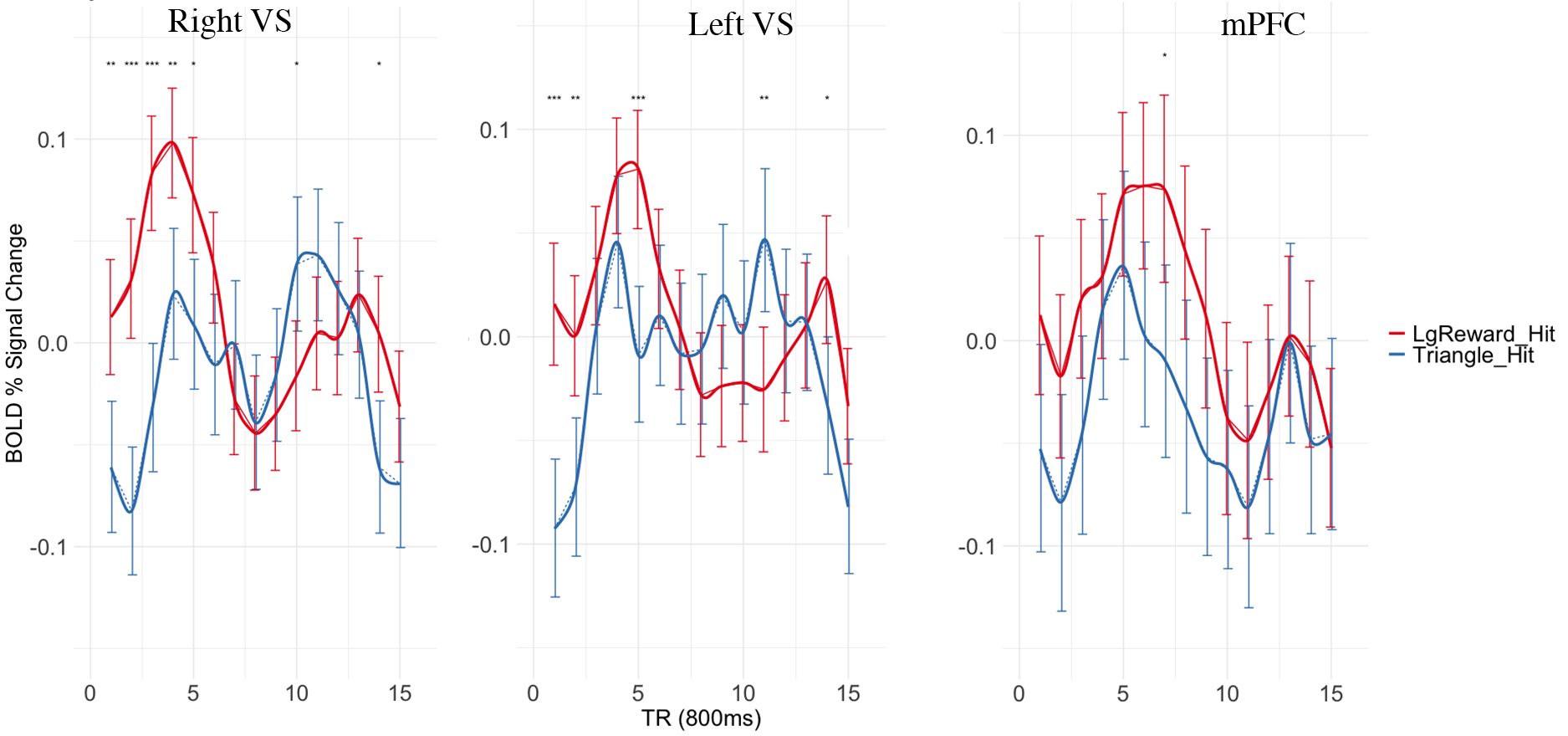


**Figure S7:** BOLD Signal Locked to Outcome onset and subsequent 15 TRs, **Big** **Win Hit versus Neutral Hit**

**LgReward Hit** [Red] = Big Win Hit; **Triangle_Hit** [Blue] = Neutral Hit. VS = Ventral Striatum; mPFC = medial prefrontal cortex.

Error bars represent 90% Confidence Interval; p < .05 * ; p < .01**, p < .001***

***Figure S8:*** BOLD Signal Locked to Outcome onset and subsequent 15 TRs, **Hit versus Miss**

**A:** LgReward Hit [Red] = Big Win Hit; Lgreward_Miss [Blue] = Big Win Miss. **B:** LgPun Hit [Red] = Big Loss Hit; LgPun_Miss [Blue] = Big Loss Miss. VS = Ventral Striatum; mPFC = medial prefrontal cortex.

Error bars represent 90% Confidence Interval; p < .05 * ; p < .01**, p < .001***


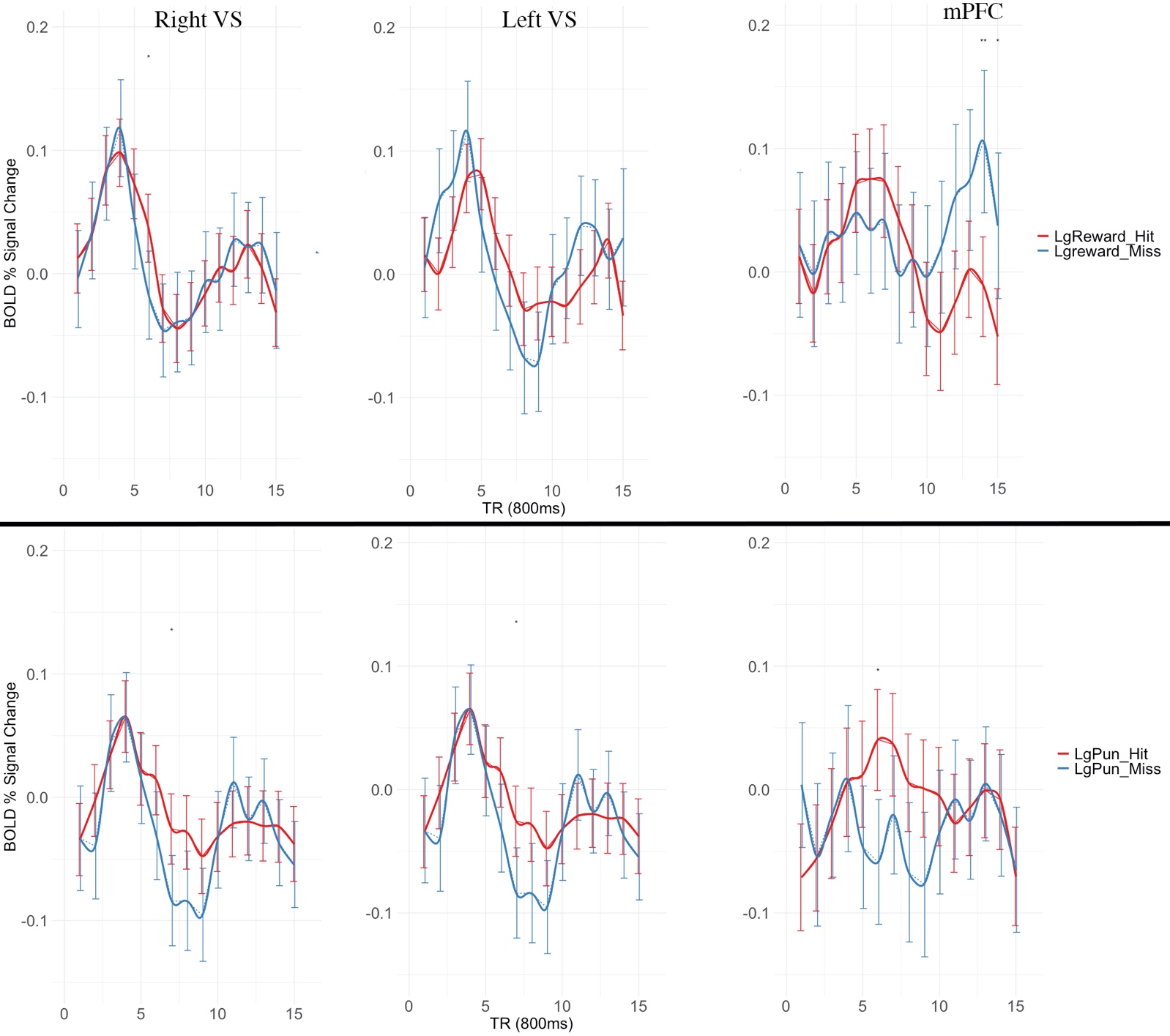


**A**

**B**

***Figure S9:*** BOLD Signal Locked to Outcome onset and subsequent 15 TRs, by **Anticipation Cue**.

**A:** LgReward [Red] = Big Win ($5) Anticipation Cue Triangle [Blue] = Neutral Anticipation Cue. **B:** LgPun [Red] = Big Loss ($5) Anticipation Cue; LgReward [Blue] = Big Win ($5) Anticipation Cue. VS = Ventral Striatum; mPFC = medial prefrontal cortex.

Error bars represent 90% Confidence Interval; p < .05 * ; p < .01**, p < .001***


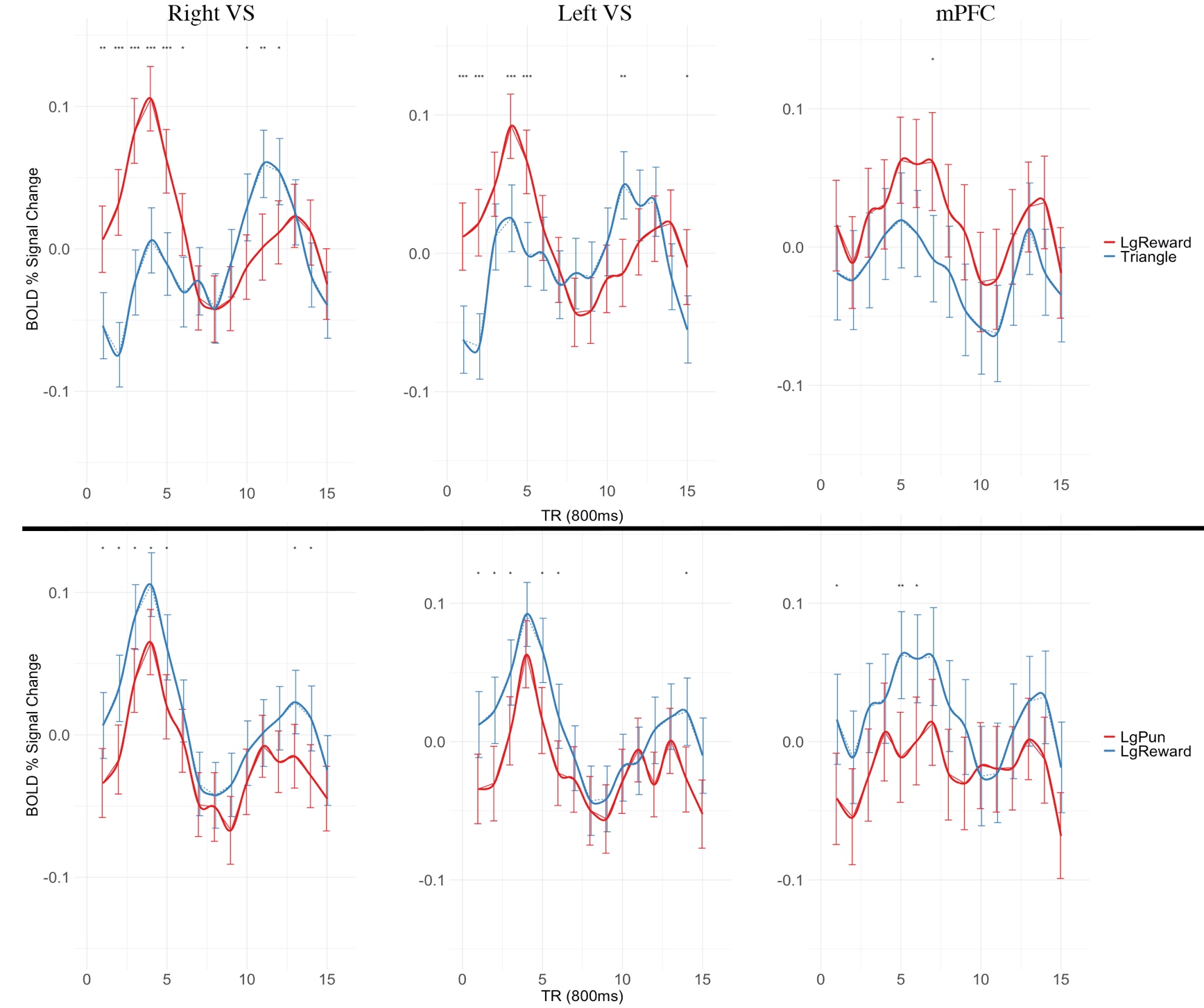


**A**

**B**

*Interpretation of the BOLD plots:*

We expand here on the interpretation of the BOLD response. The nature of the anticipation signal bleeding into the outcome phase is apparent in the bilateral VS when the anticipation cues are locked to the outcome phase (Figure S8). There is significant separation for the first 4-5 TRs (or 3-4 sec) in the outcome phase in the Big Win as compared to the Neutral phase, until they reverse by TR 10. Since the signal is not appropriately deconvolved in the outcome phase, one approach is to model based on combinations of Hit/Miss trials. In our main outcome contrasts, F6 and F7, we modeled the Big Win versus Neutral Hit, which still demonstrates poor deconvolution in the VS regions (Figure S6), which likely stems from the between contrast spill-over HRF from the anticipatory phase (Figure S5). One alternative approach, which we did not model in the whole brain contrasts, is the contrast of Big Win/Loss Hit versus Big Win/Loss Miss. However, direct observation of the BOLD signal (Figure S7) demonstrates that for Big Win Hit and Big Win Miss, there is a higher signal for hit versus miss trials, however, these are nearly identical in the VS BOLD signal. However, whereas the mPFC demonstrates peak separation at TR 14 (~11 sec), this is occurring well into the subsequent trial, it is unclear what this change represents. Overall, we find appropriate peaks in direct BOLD signal after anticipation cue onset, but a complicated picture forms in the outcome phase with respect to bilateral VS and mPFC.

*2.8 Proportion of effect sizes across* 400 brain-behavior estimates

All values are absolute values of the reported effect sizes |*r*|

**Table S6**. Count/Proportion across Pearson *r* standard effect sizes, out of 400 observations (10 contrasts x 8 ROIs x 5 behaviors)

|  | Count (n) | Proportion (%) |
| --- | --- | --- |
| Small (*r:* .00 - .20) | 388 | 97% |
| Medium (*r:* .20 - .30) | 12 | 3% |
| Large (*r* < .30 - .50) | 0 | 0% |

**Table S7.** Count/Proportion across |.05| intervals in effect sizes, out of 400 observations (rounded, may not add up exactly to 100%)

|  | Count (n) | Proportion (%) |
| --- | --- | --- |
| *r* = 0.00 to 0.049 | 164 | 41% |
| *r* = 0.05 to 0.099 | 123 | 31% |
| *r* = 0.10 to 0.149 | 72 | 18% |
| *r* = 0.15 to 0.199 | 29 | 7% |
| *r* = 0.20 to 0.249 | 11 | 3% |
| *r* = 0.25 to 0.299 | 1 | < 1% |

References

Aloi, J., Meffert, H., White, S. F., Blair, K. S., Hwang, S., Tyler, P. M., Thornton, L. C., Crum, K. I., Adams, K. O., Killanin, A. D., Filbey, F., Pope, K., & Blair, R. J. R. (2019). Differential dysfunctions related to alcohol and cannabis use disorder symptoms in reward and error-processing neuro-circuitries in adolescents. *Developmental Cognitive Neuroscience*, *36*, 100618. https://doi.org/10.1016/j.dcn.2019.100618

Boecker, R., Holz, N. E., Buchmann, A. F., Blomeyer, D., Plichta, M. M., Wolf, I., Baumeister, S., Meyer-Lindenberg, A., Banaschewski, T., Brandeis, D., & Laucht, M. (2014). Impact of early life adversity on reward processing in young adults: EEG-fMRI results from a prospective study over 25 years. *PloS One*, *9*(8), e104185. https://doi.org/10.1371/journal.pone.0104185

Bourque, J., Spechler, P. A., Potvin, S., Whelan, R., Banaschewski, T., Bokde, A. L. W., Bromberg, U., Büchel, C., Quinlan, E. B., Desrivières, S., Flor, H., Frouin, V., Gowland, P., Heinz, A., Ittermann, B., Martinot, J.-L., Paillère-Martinot, M.-L., McEwen, S. C., Nees, F., … IMAGEN Consortium. (2017). Functional Neuroimaging Predictors of Self-Reported Psychotic Symptoms in Adolescents. *The American Journal of Psychiatry*, *174*(6), 566–575. https://doi.org/10.1176/appi.ajp.2017.16080897

Büchel, C., Peters, J., Banaschewski, T., Bokde, A. L. W., Bromberg, U., Conrod, P. J., Flor, H., Papadopoulos, D., Garavan, H., Gowland, P., Heinz, A., Walter, H., Ittermann, B., Mann, K., Martinot, J.-L., Paillère-Martinot, M.-L., Nees, F., Paus, T., Pausova, Z., … Knutson, B. (2017). Blunted ventral striatal responses to anticipated rewards foreshadow problematic drug use in novelty-seeking adolescents. *Nature Communications*, *8*(1), 1–11. https://doi.org/10.1038/ncomms14140

Cao, Z., Bennett, M., Orr, C., Icke, I., Banaschewski, T., Barker, G. J., Bokde, A. L. W., Bromberg, U., Büchel, C., Quinlan, E. B., Desrivières, S., Flor, H., Frouin, V., Garavan, H., Gowland, P., Heinz, A., Ittermann, B., Martinot, J.-L., Nees, F., … Whelan, R. (2019). Mapping adolescent reward anticipation, receipt, and prediction error during the monetary incentive delay task. *Human Brain Mapping*, *40*(1), 262–283. https://doi.org/10.1002/hbm.24370

Chan, R. C. K., Li, Z., Li, K., Zeng, Y.-W., Xie, W.-Z., Yan, C., Cheung, E. F. C., & Jin, Z. (2016). Distinct processing of social and monetary rewards in late adolescents with trait anhedonia. *Neuropsychology*, *30*(3), 274–280. https://doi.org/10.1037/neu0000233

Chronaki, G., Soltesz, F., Benikos, N., & Sonuga-Barke, E. J. S. (2017). An electrophysiological investigation of reinforcement effects in attention deficit/hyperactivity disorder: Dissociating cue sensitivity from down-stream effects on target engagement and performance. *Developmental Cognitive Neuroscience*, *28*, 12–20. https://doi.org/10.1016/j.dcn.2017.10.003

Colich, N. L., Ho, T. C., Ellwood-Lowe, M. E., Foland-Ross, L. C., Sacchet, M. D., LeMoult, J. L., & Gotlib, I. H. (2017). Like mother like daughter: Putamen activation as a mechanism underlying intergenerational risk for depression. *Social Cognitive and Affective Neuroscience*, *12*(9), 1480–1489. https://doi.org/10.1093/scan/nsx073

Cope, L. M., Martz, M. E., Hardee, J. E., Zucker, R. A., & Heitzeg, M. M. (2019). Reward activation in childhood predicts adolescent substance use initiation in a high-risk sample. *Drug and Alcohol Dependence*, *194*, 318–325. https://doi.org/10.1016/j.drugalcdep.2018.11.003

Dhingra, I., Zhang, S., Zhornitsky, S., Le, T. M., Wang, W., Chao, H. H., Levy, I., & Li, C.-S. R. (2019). The effects of age on reward magnitude processing in the monetary incentive delay task. *NeuroImage*, 116368. https://doi.org/10.1016/j.neuroimage.2019.116368

Duka, T., Nikolaou, K., King, S. L., Banaschewski, T., Bokde, A. L. W., Büchel, C., Carvalho, F. M., Conrod, P. J., Flor, H., Gallinat, J., Garavan, H., Heinz, A., Jia, T., Gowland, P., Martinot, J.-L., Paus, T., Rietschel, M., Robbins, T. W., Smolka, M., … Stephens, D. N. (2017). GABRB1 Single Nucleotide Polymorphism Associated with Altered Brain Responses (but not Performance) during Measures of Impulsivity and Reward Sensitivity in Human Adolescents. *Frontiers in Behavioral Neuroscience*, *11*, 24. https://doi.org/10.3389/fnbeh.2017.00024

Garrison, K. A., Yip, S. W., Balodis, I. M., Carroll, K. M., Potenza, M. N., & Krishnan-Sarin, S. (2017). Reward-related frontostriatal activity and smoking behavior among adolescents in treatment for smoking cessation. *Drug and Alcohol Dependence*, *177*, 268–276. https://doi.org/10.1016/j.drugalcdep.2017.03.035

Gonzalez, M. Z., Allen, J. P., & Coan, J. A. (2016). Lower neighborhood quality in adolescence predicts higher mesolimbic sensitivity to reward anticipation in adulthood. *Developmental Cognitive Neuroscience*, *22*, 48–57. https://doi.org/10.1016/j.dcn.2016.10.003

Joseph, J. E., Zhu, X., Lynam, D., & Kelly, T. H. (2016). Modulation of meso-limbic reward processing by motivational tendencies in young adolescents and adults. *NeuroImage*, *129*, 40–54. https://doi.org/10.1016/j.neuroimage.2015.12.005

Karoly, H. C., Bryan, A. D., Weiland, B. J., Mayer, A., Dodd, A., & Feldstein Ewing, S. W. (2015). Does incentive-elicited nucleus accumbens activation differ by substance of abuse? An examination with adolescents. *Developmental Cognitive Neuroscience*, *16*, 5–15. https://doi.org/10.1016/j.dcn.2015.05.005

Landes, I., Bakos, S., Kohls, G., Bartling, J., Schulte-Körne, G., & Greimel, E. (2018). Altered neural processing of reward and punishment in adolescents with Major Depressive Disorder. *Journal of Affective Disorders*, *232*, 23–33. https://doi.org/10.1016/j.jad.2018.01.017

LeMoult, J., Colich, N. L., Sherdell, L., Hamilton, J. P., & Gotlib, I. H. (2015). Influence of menarche on the relation between diurnal cortisol production and ventral striatum activity during reward anticipation. *Social Cognitive and Affective Neuroscience*, *10*(9), 1244–1250. https://doi.org/10.1093/scan/nsv016

Li, Z., Yan, C., Xie, W.-Z., Li, K., Zeng, Y.-W., Jin, Z., Cheung, E. F. C., & Chan, R. C. K. (2015). Anticipatory pleasure predicts effective connectivity in the mesolimbic system. *Frontiers in Behavioral Neuroscience*, *9*, 217. https://doi.org/10.3389/fnbeh.2015.00217

Maresh, E. L., Stim, J. J., Van Voorhis, A. C., Kang, S. S., Luciana, M., Sponheim, S. R., & Urošević, S. (2019). Neurophysiological correlates of cognitive control and approach motivation abnormalities in adolescent bipolar disorders. *Cognitive, Affective & Behavioral Neuroscience*, *19*(3), 677–691. https://doi.org/10.3758/s13415-019-00719-x

Martz, M. E., Trucco, E. M., Cope, L. M., Hardee, J. E., Jester, J. M., Zucker, R. A., & Heitzeg, M. M. (2016). Association of Marijuana Use With Blunted Nucleus Accumbens Response to Reward Anticipation. *JAMA Psychiatry*, *73*(8), 838–844. https://doi.org/10.1001/jamapsychiatry.2016.1161

Martz, M. E., Zucker, R. A., Schulenberg, J. E., & Heitzeg, M. M. (2018). Psychosocial and neural indicators of resilience among youth with a family history of substance use disorder. *Drug and Alcohol Dependence*, *185*, 198–206. https://doi.org/10.1016/j.drugalcdep.2017.12.015

Mikita, N., Simonoff, E., Pine, D. S., Goodman, R., Artiges, E., Banaschewski, T., Bokde, A. L., Bromberg, U., Büchel, C., Cattrell, A., Conrod, P. J., Desrivières, S., Flor, H., Frouin, V., Gallinat, J., Garavan, H., Heinz, A., Ittermann, B., Jurk, S., … Stringaris, A. (2016). Disentangling the autism-anxiety overlap: FMRI of reward processing in a community-based longitudinal study. *Translational Psychiatry*, *6*(6), e845. https://doi.org/10.1038/tp.2016.107

Mori, A., Okamoto, Y., Okada, G., Takagaki, K., Jinnin, R., Takamura, M., Kobayakawa, M., & Yamawaki, S. (2016). Behavioral activation can normalize neural hypoactivation in subthreshold depression during a monetary incentive delay task. *Journal of Affective Disorders*, *189*, 254–262. https://doi.org/10.1016/j.jad.2015.09.036

Navas, J. F., Barrós-Loscertales, A., Costumero-Ramos, V., Verdejo-Román, J., Vilar-López, R., & Verdejo-García, A. (2018). Excessive body fat linked to blunted somatosensory cortex response to general reward in adolescents. *International Journal of Obesity (2005)*, *42*(1), 88–94. https://doi.org/10.1038/ijo.2017.207

Nees, F., Witt, S. H., Dinu-Biringer, R., Lourdusamy, A., Tzschoppe, J., Vollstädt-Klein, S., Millenet, S., Bach, C., Poustka, L., Banaschewski, T., Barker, G. J., Bokde, A. L. W., Bromberg, U., Büchel, C., Conrod, P. J., Frank, J., Frouin, V., Gallinat, J., Garavan, H., … IMAGEN consortium. (2015). BDNF Val66Met and reward-related brain function in adolescents: Role for early alcohol consumption. *Alcohol (Fayetteville, N.Y.)*, *49*(2), 103–110. https://doi.org/10.1016/j.alcohol.2014.12.004

Nestor, L. J., Behan, B., Suckling, J., & Garavan, H. (2019). Cannabis-dependent adolescents show differences in global reward-associated network topology: A functional connectomics approach. *Addiction Biology*, e12752. https://doi.org/10.1111/adb.12752

Papanastasiou, E., Mouchlianitis, E., Joyce, D. W., McGuire, P., Banaschewski, T., Bokde, A. L. W., Bromberg, U., Büchel, C., Quinlan, E. B., Desrivières, S., Flor, H., Frouin, V., Garavan, H., Spechler, P., Gowland, P., Heinz, A., Ittermann, B., Martinot, J.-L., Paillère Martinot, M.-L., … IMAGEN Consortium. (2018). Examination of the Neural Basis of Psychoticlike Experiences in Adolescence During Reward Processing. *JAMA Psychiatry*, *75*(10), 1043–1051. https://doi.org/10.1001/jamapsychiatry.2018.1973

Richards, J. S., Arias Vásquez, A., von Rhein, D., van der Meer, D., Franke, B., Hoekstra, P. J., Heslenfeld, D. J., Oosterlaan, J., Faraone, S. V., Buitelaar, J. K., & Hartman, C. A. (2016). Adolescent behavioral and neural reward sensitivity: A test of the differential susceptibility theory. *Translational Psychiatry*, *6*, e771. https://doi.org/10.1038/tp.2016.37

Sauder, C. L., Derbidge, C. M., & Beauchaine, T. P. (2016). Neural responses to monetary incentives among self-injuring adolescent girls. *Development and Psychopathology*, *28*(1), 277–291. https://doi.org/10.1017/S0954579415000449

Schwartz, K. T. G., Kryza-Lacombe, M., Liuzzi, M. T., Weersing, V. R., & Wiggins, J. L. (2019). Social and Non-social Reward: A Preliminary Examination of Clinical Improvement and Neural Reactivity in Adolescents Treated With Behavioral Therapy for Anxiety and Depression. *Frontiers in Behavioral Neuroscience*, *13*. https://doi.org/10.3389/fnbeh.2019.00177

Stevens, M. C., Pearlson, G. D., Calhoun, V. D., & Bessette, K. L. (2018). Functional Neuroimaging Evidence for Distinct Neurobiological Pathways in Attention-Deficit/Hyperactivity Disorder. *Biological Psychiatry. Cognitive Neuroscience and Neuroimaging*, *3*(8), 675–685. https://doi.org/10.1016/j.bpsc.2017.09.005

Swartz, J. R., Weissman, D. G., Ferrer, E., Beard, S. J., Fassbender, C., Robins, R. W., Hastings, P. D., & Guyer, A. E. (2019). Reward-Related Brain Activity Prospectively Predicts Increases in Alcohol Use in Adolescents. *Journal of the American Academy of Child and Adolescent Psychiatry*. https://doi.org/10.1016/j.jaac.2019.05.022

Urošević, S., Luciana, M., Jensen, J. B., Youngstrom, E. A., & Thomas, K. M. (2016). Age associations with neural processing of reward anticipation in adolescents with bipolar disorders. *NeuroImage. Clinical*, *11*, 476–485. https://doi.org/10.1016/j.nicl.2016.03.013

van Hulst, B. M., de Zeeuw, P., Lupas, K., Bos, D. J., Neggers, S. F. W., & Durston, S. (2015). Reward Anticipation in Ventral Striatum and Individual Sensitivity to Reward: A Pilot Study of a Child-Friendly fMRI Task. *PloS One*, *10*(11), e0142413. https://doi.org/10.1371/journal.pone.0142413

Veroude, K., von Rhein, D., Chauvin, R. J. M., van Dongen, E. V., Mennes, M. J. J., Franke, B., Heslenfeld, D. J., Oosterlaan, J., Hartman, C. A., Hoekstra, P. J., Glennon, J. C., & Buitelaar, J. K. (2016). The link between callous-unemotional traits and neural mechanisms of reward processing: An fMRI study. *Psychiatry Research. Neuroimaging*, *255*, 75–80. https://doi.org/10.1016/j.pscychresns.2016.08.005

von Rhein, D., Cools, R., Zwiers, M. P., van der Schaaf, M., Franke, B., Luman, M., Oosterlaan, J., Heslenfeld, D. J., Hoekstra, P. J., Hartman, C. A., Faraone, S. V., van Rooij, D., van Dongen, E. V., Lojowska, M., Mennes, M., & Buitelaar, J. (2015). Increased neural responses to reward in adolescents and young adults with attention-deficit/hyperactivity disorder and their unaffected siblings. *Journal of the American Academy of Child and Adolescent Psychiatry*, *54*(5), 394–402. https://doi.org/10.1016/j.jaac.2015.02.012

Xu, B., Jia, T., Macare, C., Banaschewski, T., Bokde, A. L. W., Bromberg, U., Büchel, C., Cattrell, A., Conrod, P. J., Flor, H., Frouin, V., Gallinat, J., Garavan, H., Gowland, P., Heinz, A., Ittermann, B., Martinot, J.-L., Paillère Martinot, M.-L., Nees, F., … IMAGEN Consortium. (2017). Impact of a Common Genetic Variation Associated With Putamen Volume on Neural Mechanisms of Attention-Deficit/Hyperactivity Disorder. *Journal of the American Academy of Child and Adolescent Psychiatry*, *56*(5), 436-444.e4. https://doi.org/10.1016/j.jaac.2017.02.009
